# miR-7a-5p attenuates hypoxic-ischemic encephalopathy by suppressing PARP1-mediated parthanatos

**DOI:** 10.1016/j.isci.2026.117947

**Published:** 2026-10-27

**Authors:** Danmei Chen, Xiangyu Kong, Hequan Zhong, Yunpeng Zhang, Chenqin Si, Ayipaxiaguli Kasimu, Rui Qiao, Yuting Song, Bing Li

**Affiliations:** 1 Department of Traditional Chinese Medicine, Jinshan Hospital of Fudan University, Shanghai 201508, China; 2 Research Center for Clinical Medicine, Jinshan Hospital of Fudan University, Shanghai 201508, China; 3 Department of Neurology, Jinshan Hospital of Fudan University, Shanghai 201508, China; 4 College of Acupuncture-Massage and Rehabilitation, Yunnan University of Traditional Chinese Medicine, Kunming, Yunnan 650500, China

**Keywords:** hypoxic-ischemic encephalopathy, miR-7a-5p, PARP1, parthanatos, neuronal injury

## Abstract

Hypoxic-ischemic encephalopathy (HIE) causes severe neonatal brain injury, with PARP1 hyperactivation implicated in neuronal death. Using a rat HIE model and glutamate-exposed HT22 neurons, we found that miR-7a-5p is significantly downregulated in both peripheral blood and the injured cortex of HIE rats. PARP1 hyperactivation triggered parthanatos, characterized by PAR polymer accumulation and apoptosis-inducing factor (AIF) nuclear translocation, without activating caspase-dependent apoptosis. Luciferase assays confirmed direct targeting of miR-7a-5p to the 3′ UTR of PARP1, suppressing its expression. Exogenous miR-7a-5p reduces infarction volume, improves neural morphology, and preserves neurological function recovery in HIE rats. *In vitro*, miR-7a-5p overexpression or pharmacological PARP1 inhibition attenuated glutamate-induced neuronal death, mitochondrial membrane potential (MMP) loss, ROS overproduction, and PAR/AIF dysregulation. Crucially, PARP1 agonist MNNG reversed miR-7a-5p-mediated neuroprotection, establishing the indispensability of the miR-7a-5p/PARP1 axis. miR-7a-5p is a key regulator of parthanatos, suppressing PARP1 hyperactivation, mitigating mitochondrial dysfunction, and offering a therapeutic target for HIE.

## Highlights

• miR-7a-5p targets PARP1 and is downregulated in HIE
• miR-7a-5p inhibits PARP1 hyperactivation and parthanatos
• miR-7a-5p attenuates mitochondrial dysfunction and ROS
• miR-7a-5p promotes recovery and is a therapeutic target for HIE

## Graphical abstract

**Figure:**
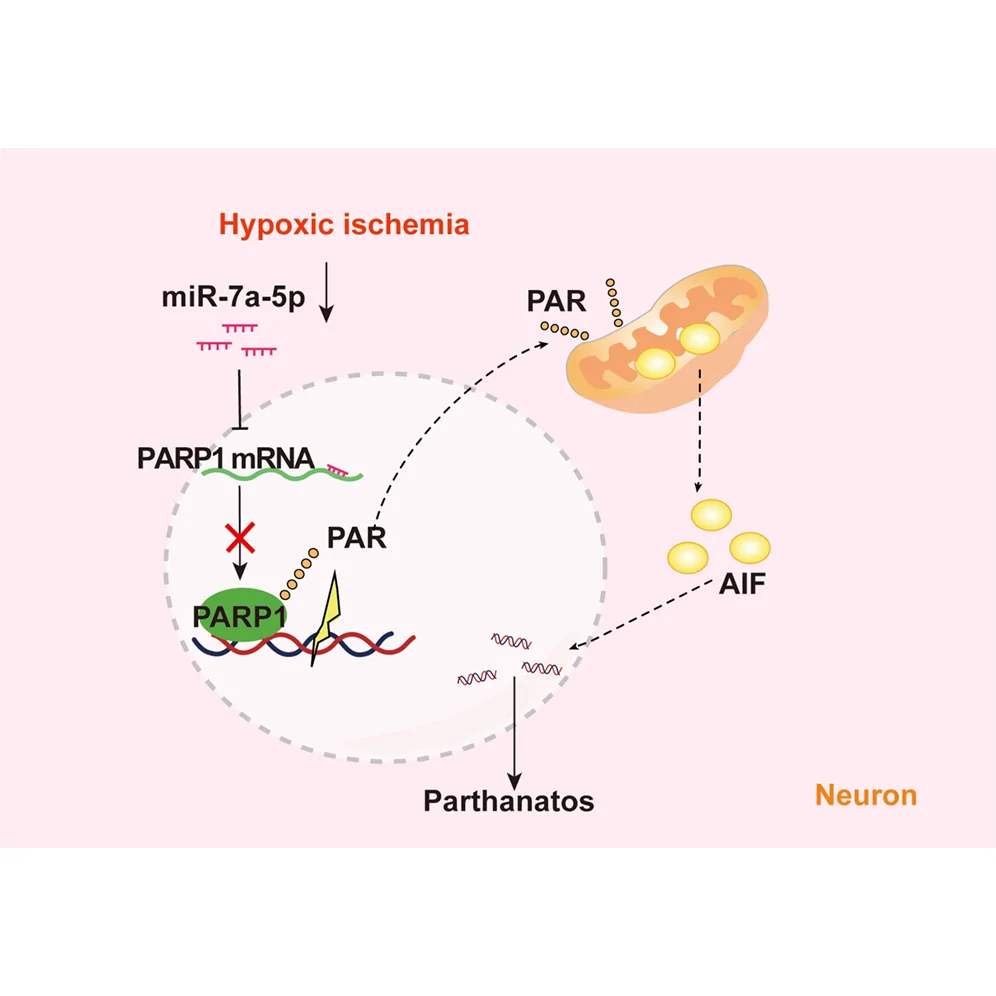

## Teaser

Biological sciences

## Introduction

Hypoxic-ischemic encephalopathy (HIE), a severe brain injury caused by perinatal hypoxia and ischemia, is a major cause of neonatal mortality and long-term neurodevelopmental impairments. Globally, it affects 1–8 per 1,000 live births.^1^^,^^2^ Approximately 15% of affected newborns succumb within the first week of life, and 40% of survivors suffer from permanent neurological sequelae, such as cerebral palsy, cognitive impairment, sensory disorders, behavioral disorders, and epilepsy.^3^^,^^4^

MicroRNAs (miRNAs) are key post-transcriptional regulators critically involved in the pathophysiology of perinatal brain injury and HIE.^5^ It was found that miR-499-5p has a neuroprotective effect by reducing neuronal cell apoptosis in a rat model of HIE.^6^ miR-210 is upregulated in response to hypoxic conditions and contributes to mitochondrial dysfunction and neuronal death.^7^ Another miRNA of interest is miR-146b-5p, which has been shown to protect oligodendrocyte precursor cells from injury induced by oxygen and glucose deprivation.^8^ These findings highlight miRNAs as key therapeutic targets in perinatal brain injury and HIE. Modulating their expression to regulate key molecular pathways could pave the way for novel therapies in neonates.

Our bioinformatics analysis of the GEO dataset GSE121178 revealed miR-7-2 as a significantly downregulated miRNA in whole blood samples from patients with HIE. The precursor gene miR-7-2 undergoes post-transcriptional processing to generate the biologically active mature miR-7a-5p, which then exerts its target-specific regulatory functions. miR-7a-5p has been closely associated with hypoxic-ischemic brain injury, and its differential expression pattern has been detected in blood samples from patients with acute large vessel occlusion stroke.^9^ Furthermore, miR-7a-5p has been found to be expressed at lower levels in the brain of stroke rats and has been verified to alleviate cardiomyocyte damage induced by hypoxia-ischemia.^10^ While its role in mitigating myocardial ischemic injury and altered expression in stroke models are established, the functional significance of miR-7a-5p in hypoxic-ischemic injury during HIE remains uncharacterized.

Among the many target genes of miR-7a-5p, poly (ADP-ribose) polymerase-1 (PARP1) plays a critical role in DNA damage repair.^11^ However, its overactivation results in nicotinamide adenine dinucleotide (NAD+) depletion, mitochondrial release of apoptosis-inducing factor (AIF),^12^ and nuclear translocation of AIF/macrophage migration inhibitory factor (MIF), initiating parthanatos^13^—a cell death pathway strongly linked to cerebral ischemia and neurodegenerative diseases.^14^^,^^15^^,^^16^ Parthanatos is a caspase-independent and PARP1-mediated programmed cell death. It was first reported as early as 1997 that PARP1 could mediate cerebral ischemic injury.^17^ Recent studies have demonstrated that 4-*O*-methylbavachalcone alleviates ischemic stroke injury by targeting SIRT3 to suppress parthanatos-related proteins (PARP1, PAR, and AIF).^18^ Although parthanatos was first linked to ischemic brain injury over two decades ago, its contribution to HIE pathology remains underexplored, particularly in the context of miRNA regulation.

To address these gaps, we investigated two interconnected hypotheses: parthanatos dominates neuronal death in HIE, and the miR-7a-5p/PARP1 axis modulates this process under hypoxic-ischemic conditions. By combining *in vivo* HIE animal models and *in vitro* cellular systems, we elucidated the central pathological role of parthanatos in HIE. Furthermore, we confirmed the neuroprotective effects of the miR-7a-5p/PARP1 axis through parthanatos suppression, offering a novel miRNA-based therapeutic avenue for neonatal brain injury.

## Results

### Differential miRNA expression profiles in HIE patients

Microarray data of human whole blood from 3 HIE patients and 3 normal controls were extracted from GSE121178 dataset. A boxplot was used to show the inter-sample differences after normalization (Figure 1A). The two-dimensional principal component analysis (PCA) cluster diagram showed optimal clustering after eliminating inter-sample differences (Figure 1B), indicating that the sample source was reliable. In total, 40 miRNAs between the HIE and control groups were extracted from the gene expression matrix based on the criteria |log2FC| > 1 and *p* value <0.05. The genes were visualized in a volcano plot (Figure 1C). The heatmap showed that the expression of these genes differed significantly between the two groups (Figure 1D). Among these, miR-7-2 was significantly downregulated in HIE patients compared to controls (Table S1). On the basis of a thorough literature review, miR-7-2 was identified as the candidate for further study (Table S2).

**Figure 1:**
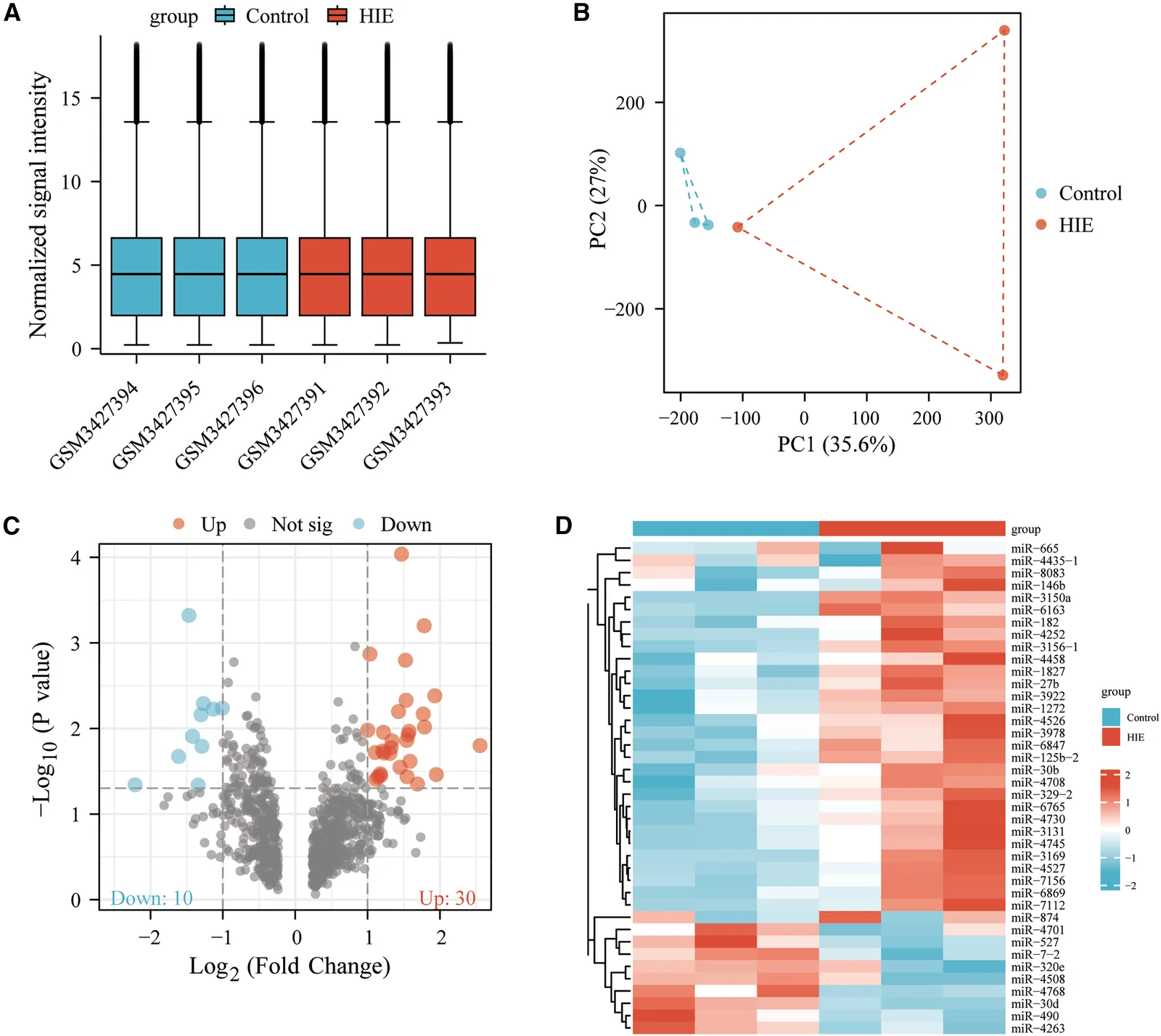
Expression analysis of miRNAs based on microarray data derived from human whole blood (A) A boxplot was used to show the inter-sample differences after normalization. (B) The two-dimensional PCA cluster diagram showed optimal clustering after eliminating inter-sample differences. (C) The volcano plot exhibited the differentially expressed miRNAs. (D) The heatmap revealed significant differences in the expression levels of these miRNAs between the two groups.

### Hypoxia-ischemia impaired the motor function of HIE rats

The righting reflex experiment showed that from P9 to P11, the rats in the model group took longer time to right their position than those in the control group (Figure 2A). The inclined plane test showed the maximum angle at which rats in the model group could remain still was smaller than that in the control group (Figure 2B). To determine whether motor and balance performance declined after hypoxia-ischemia (HI), we conducted the balance beam test. HIE rats took longer time to cross the beam compared with sham controls, and the hind limbs of HIE rats slipped more than those of sham rats’ when crossing the beam (Figures 2C and 2D).

**Figure 2:**
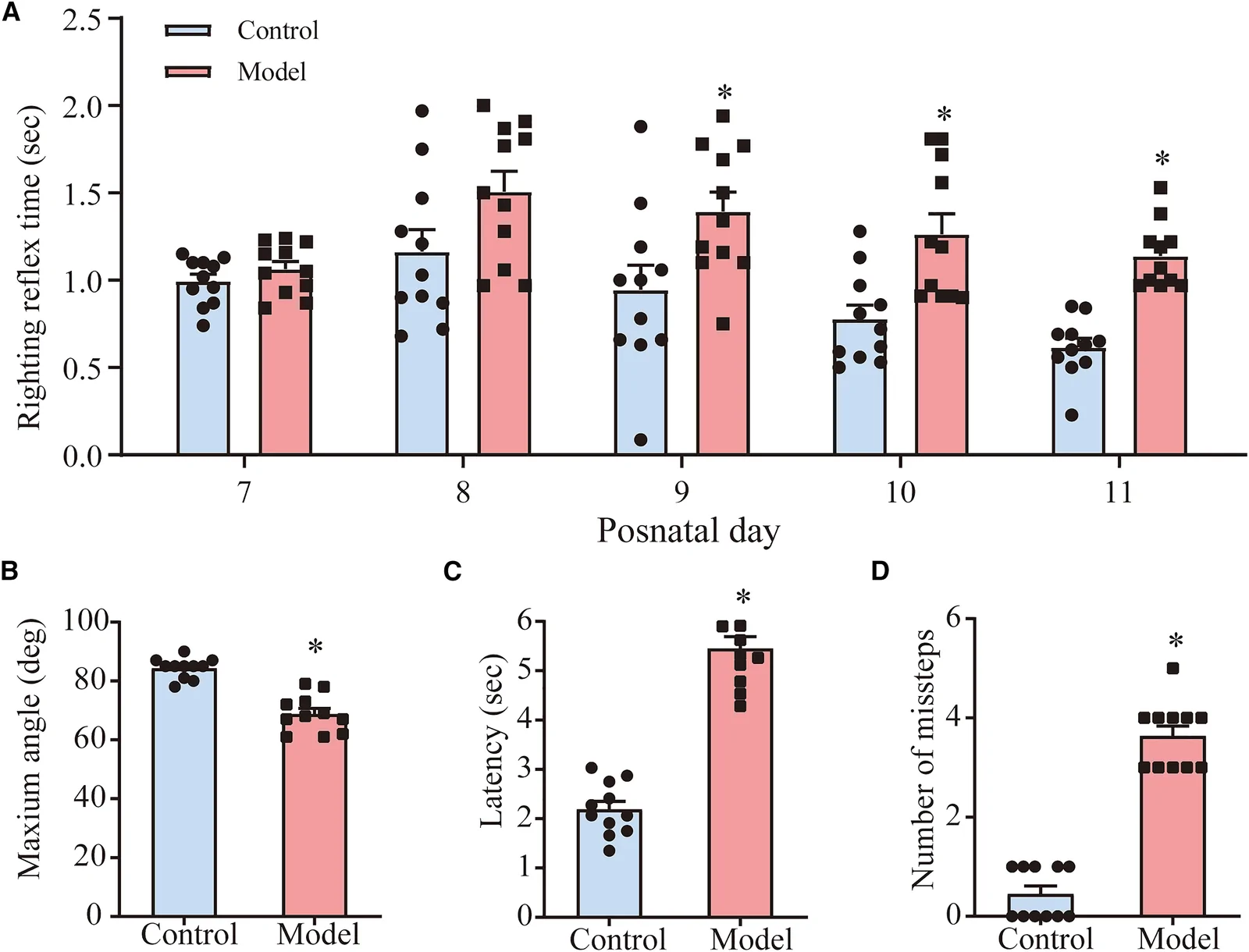
HI dampened motor function of HIE rats (A) Time required for the rats to right their position. (B) Maximum angle of the inclined plane test that rats can stay still. (C) Time required for the rats to cross the beam. (D) Number of slips of the hind limbs while crossing the beam. Data are expressed as mean (±SD, *n* = 11 individual animals per group) and analyzed for statistical significance using a *t* test (∗*p* < 0.05 versus the control group).

### miR-7a-5p targets PARP1 and parthanatos dominates neuronal death in HIE

Real-time PCR showed that the expression level of miR-7a-5p was downregulated in the injured cortex and peripheral blood of HIE rats (Figures 3A and 3B). According to the TargetScan database, miR-7a-5p is predicted to potentially bind to the nucleotide sequence positions 628–635 within the 3′ untranslated region (3′ UTR) of PARP1 mRNA. Further experiments validated the binding potential between miR-7a-5p and PARP1: the miR-7a-5p mimic suppressed the luciferase activity of PARP1 wild-type HEK293T cells, whereas had no effect on PARP1-mutated HEK293T cells (Figures 3C and 3D). Western blotting indicated that PARP1 was hyperactivated in the injured cortex of HIE rats (Figures 3E and 3F). PARP1 plays roles in both apoptosis and parthanatos, and cleaved-PARP1 is reported as one of the effectors and markers of apoptosis. However, western blotting analysis showed that the expression of cleaved-PARP1 exhibited no difference between the control group and the HIE group (Figure 3E), whereas ,the expression of PAR and AIF, downstream proteins of parthanatos, were higher in the cortex of HIE rats in comparison with the control group (Figures 3E, 3G, and 3H). Electron microscopy showed that the number of mitochondria within neurons of the injured cortex of HIE rats decreased, and they appeared swollen, with disrupted cristae, consistent with parthanatos-driven damage (Figure 3I). Taken together, miR-7a-5p was significantly downregulated in HIE rats, and its suppression of PARP1 via binding to the 3′ UTR of PARP1 was validated. PARP1 hyperactivation triggered parthanatos, as evidenced by elevated PAR and AIF levels, mitochondrial damage, and unchanged apoptosis markers (cleaved-PARP1), indicating that parthanatos is the dominant neuronal death pathway in HIE.

**Figure 3:**
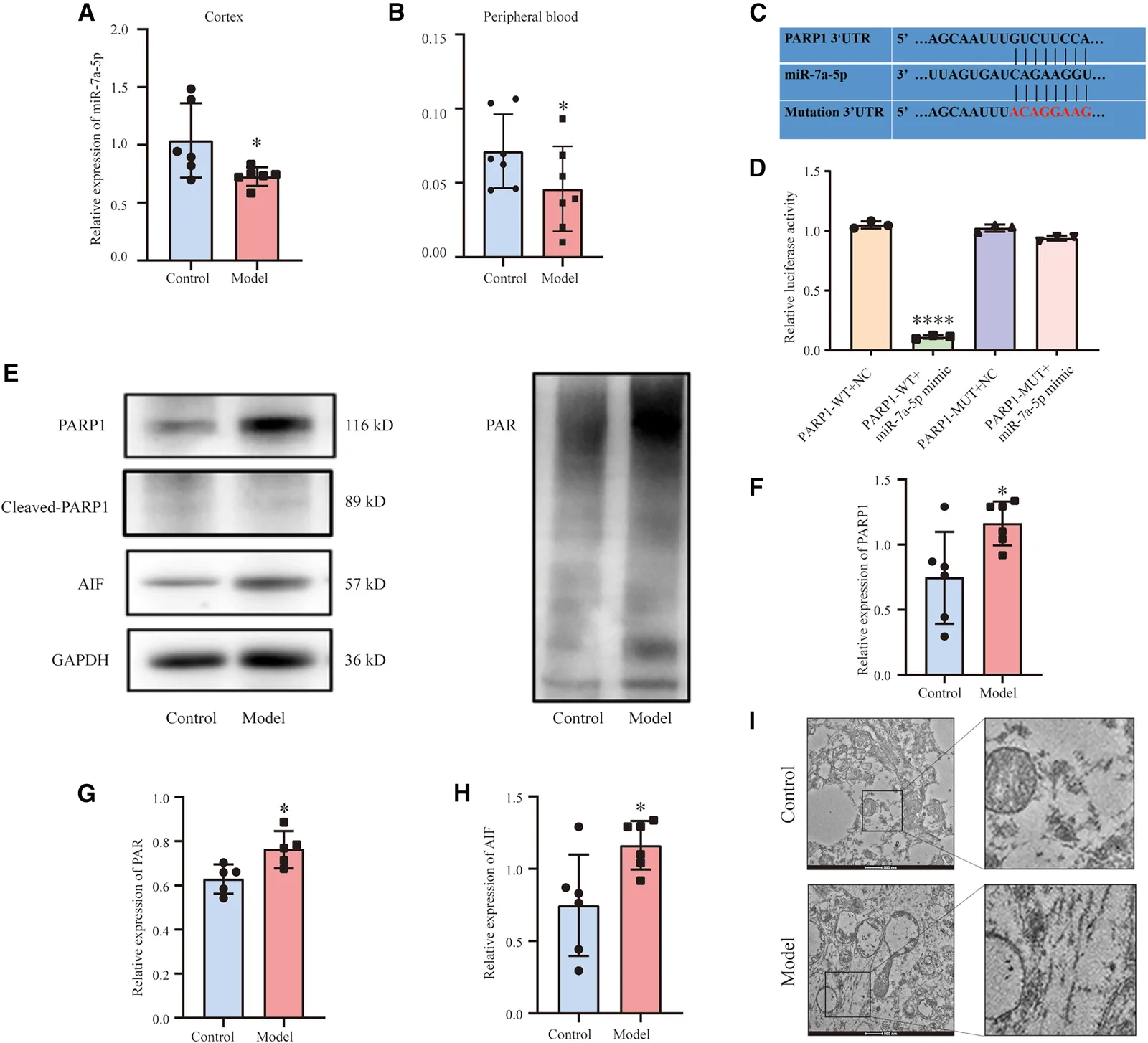
The miR-7a-5p could regulate the expression of parthanatos initiator PARP1 and HI-induced parthanatos within the cortex (A and B) The expression levels of miR-7a-5p in peripheral blood (*n* = 7, individual animals per group) and cortex (*n* = 6, individual animals per group) of HIE rats. (C) A prediction of binding sites between miR-7a-5p and PARP1. (D) Luciferase reporter gene assay validating the interaction between miR-7a-5p and PARP1 (*n* = 3, independent repeats). The data are expressed as mean ± SD and analyzed for statistical significance using one-way ANOVA (∗*p* < 0.05 versus PARP1-WT + NC group). (E–H) Western blotting analysis showed the expression of PARP1, AIF (*n* = 6, individual animals per group) and PAR (*n* = 6, individual animals per group) in the cortex of rats. The data are expressed as mean ± SD and analyzed for statistical significance using a *t* test (∗*p* < 0.05 versus the control group). (I) Representative electron micrograph of mitochondria in two groups (*n* = 3, individual animals per group). Scale bars, 1 μm.

### The miR-7a-5p AG alleviated brain damage and improved brain function in HI rats

To investigate the potential neuroprotective effects of miR-7a-5p in neonatal HI injury, miR-7a-5p agomir (AG) was administered via intracerebroventricular (i.c.v.) injection at 6h post-HI. 2,3,5-Triphenyltetrazolium chloride (TTC) staining performed at 48 h post-HI revealed that miR-7a-5p AG significantly reduced cerebral infarct volume in HI rats compared to the model + negative control (NC) group (Figure 4A). Electron microscopy further demonstrated that miR-7a-5p AG reduced mitochondrial damage and chromatin condensation in cortical neurons of HIE rats (Figure 4B). Additionally, the novel object recognition (NOR) test results revealed that the model + NC group spent a significantly lower percentage of time exploring the novel object compared to the control group, whereas the model + AG group spent a significantly higher percentage than the model + NC group (Figures 4C and 4D). Collectively, these results demonstrate that miR-7a-5p reduces cerebral infarction and enhances neurological recovery, particularly recognition memory, after HI injury.

**Figure 4:**
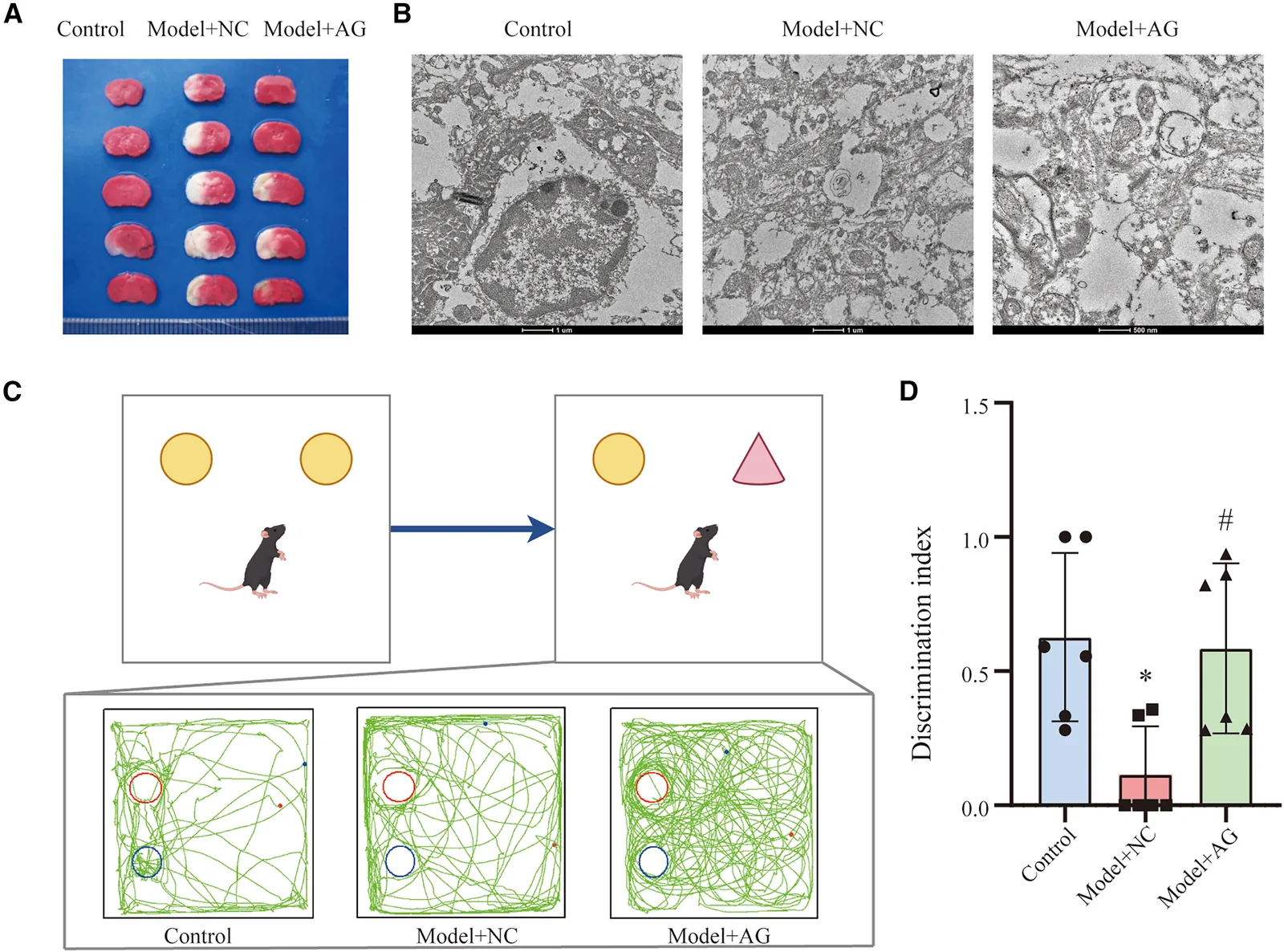
The miR-7a-5p agomir alleviated brain damage and improved brain function in HI rats (A) Representative image of TTC staining in the three groups (*n* = 3, individual animals per group). (B) Representative electron micrograph of mitochondria and nuclear chromatin in three groups (*n* = 3, individual animals per group). (C) Movement trajectories of rats in the three groups during the novel object recognition test. (D) Percentage of time spent exploring the novel object in the three groups (*n* = 6, individual animals per group). The data are expressed as mean ± SD and analyzed for statistical significance using one-way ANOVA (∗*p* < 0.05 versus the control group, #*p* < 0.05 versus the model + NC group).

### L-glutamate triggered neuronal parthanatos in HT22 cells

To further study the role of parthanatos in HIE, we utilized L-glutamate to induce oxidative stress in HT22 cells. Flow cytometric analysis also showed that a 12 h incubation of 5 mM L-glutamate could effectively induce cell death of HT22 cells (Figures 5A, 5B, and 5D). 5,5′,6,6′-tetrachloro-1,1′,3,3′-tetraethylbenzimidazolylcarbocyanine iodide (JC-1) staining suggested that MMP significantly decreased in L-glutamate-treated HT22 cells (Figure 5F). The 2′,7′-dichlorofluorescein-diacetate (DCFH-DA) probe indicated that the L-glutamate-treated HT22 cells contained higher levels of reactive oxygen species (ROS) (Figure 5E). Considering the substantial ROS production, we sought to determine whether ferroptosis or necroptosis was concurrently active. Western blotting analysis revealed no significant differences in the protein levels of GPX4 or ACSL4 between the two groups. And the necroptotic markers p-RIP3 and p-MLKL showed no significant increase in L-glutamate-treated HT22 cells (Figures S1A and S1B). These findings were further validated in the cerebral cortex tissue of HIE rats (Figures S1C and S1D). Consistent with findings in animal cortex samples, we also found that the expression of miR-7a-5p was decreased in L-glutamate-treated HT22 cells (Figure 5C). The expression of cleaved-PARP1 was quite low in both groups, whereas the expression of PARP1 was higher after L-glutamate incubation. Moreover, the expression levels of PARP1, PAR, and AIF were higher in total cell lysis of L-glutamate-treated HT22 cells (Figures 5G and 5I). The nuclei of L-glutamate-treated HT22 cells contained higher levels of AIF and MIF compared to control HT22 cells (Figures 5H, 5J, and 5K). Taken together, L-glutamate induced oxidative stress-triggered parthanatos in HT22 cells.

**Figure 5:**
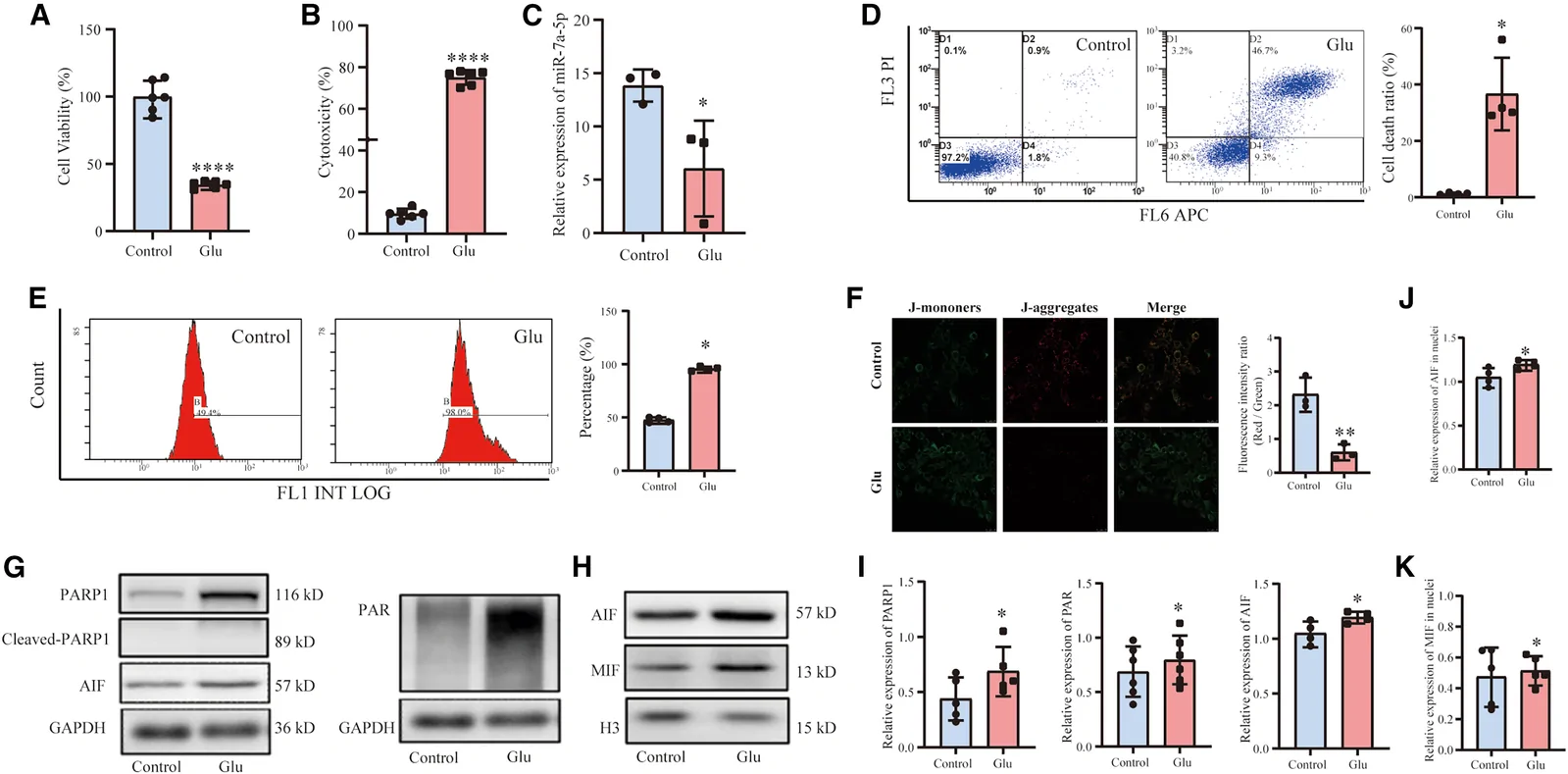
Glutamate-induced cell injury and mitochondrial dysfunction in HT22 cells (A and B) Cell viability and cytotoxicity of HT22 cells (*n* = 6, independent repeats). (C) The expression level of miR-7a-5p (*n* = 3, independent repeats). (D) Cell death ratio of HT22 cells determined by flow cytometric analysis (*n* = 4, independent repeats). (E) ROS production of HT22 cells determined by flow cytometric analysis (*n* = 4, independent repeats). (F) Mitochondrial membrane potentials in HT22 cells detected by JC-1 staining (*n* = 3, independent repeats). (G–K) Western blotting analysis showed the expression of PARP1 (*n* = 6, independent repeats), AIF (*n* = 4, independent repeats) and PAR (*n* = 6, independent repeats) in whole cell lysate (G and I) and expression of AIF (*n* = 4, independent repeats), MIF (*n* = 6, independent repeats) in the nuclei extract (H, J, and K). The data are expressed as mean ± SD and analyzed for statistical significance using one-way ANOVA (∗*p* < 0.05, ∗∗∗*p* < 0.001, ∗∗∗∗*p* < 0.0001 versus the control group).

### Pharmacological inhibition of PARP1 attenuates parthanatos in L-glutamate-induced neuronal oxidative stress

To investigate the central role of parthanatos in HIE, we utilized N-(6-oxo-5,6-dihydro-henanthridin-2-yl)-N,N-dimethylacetamide HCl hydrochloride hydrate (PJ34) to inhibit PARP1. L-glutamate-induced cell death can be attenuated by several concentrations of PJ34, and the protective effect reached a peak at 320 μM, which was applied in the following experiments (Figure 6A). Flow cytometry also showed that PJ34 can attenuate L-glutamate-induced cell death (Figure 6D). Since excessive ATP consumption is a defining characteristic of parthanatos, we also examined how the interventions affected cellular energy consumption. It was found that PJ34 not only reduced the ROS levels caused by L-glutamate incubation, but also alleviated the excessive consumption of ATP (Figures 6E and S2). L-glutamate also induced significant PAR formation, which was significantly reduced by PJ34. As one of the most important downstream effective molecules, AIF showed a higher expression level in whole cell lysis in the Glu group (Figures 6B and 6C). After L-glutamate incubation, the expression of PAR was significantly increased (Figure 6F), and the Mander’s coefficient of AIF in the nuclei was also augmented (Figure 6G). Critically, all these effects—PAR formation, AIF nuclear translocation, and ROS overproduction—were reversed by PJ34. This demonstrates that parthanatos is a critical pathological outcome of excitotoxicity in HIE.

**Figure 6:**
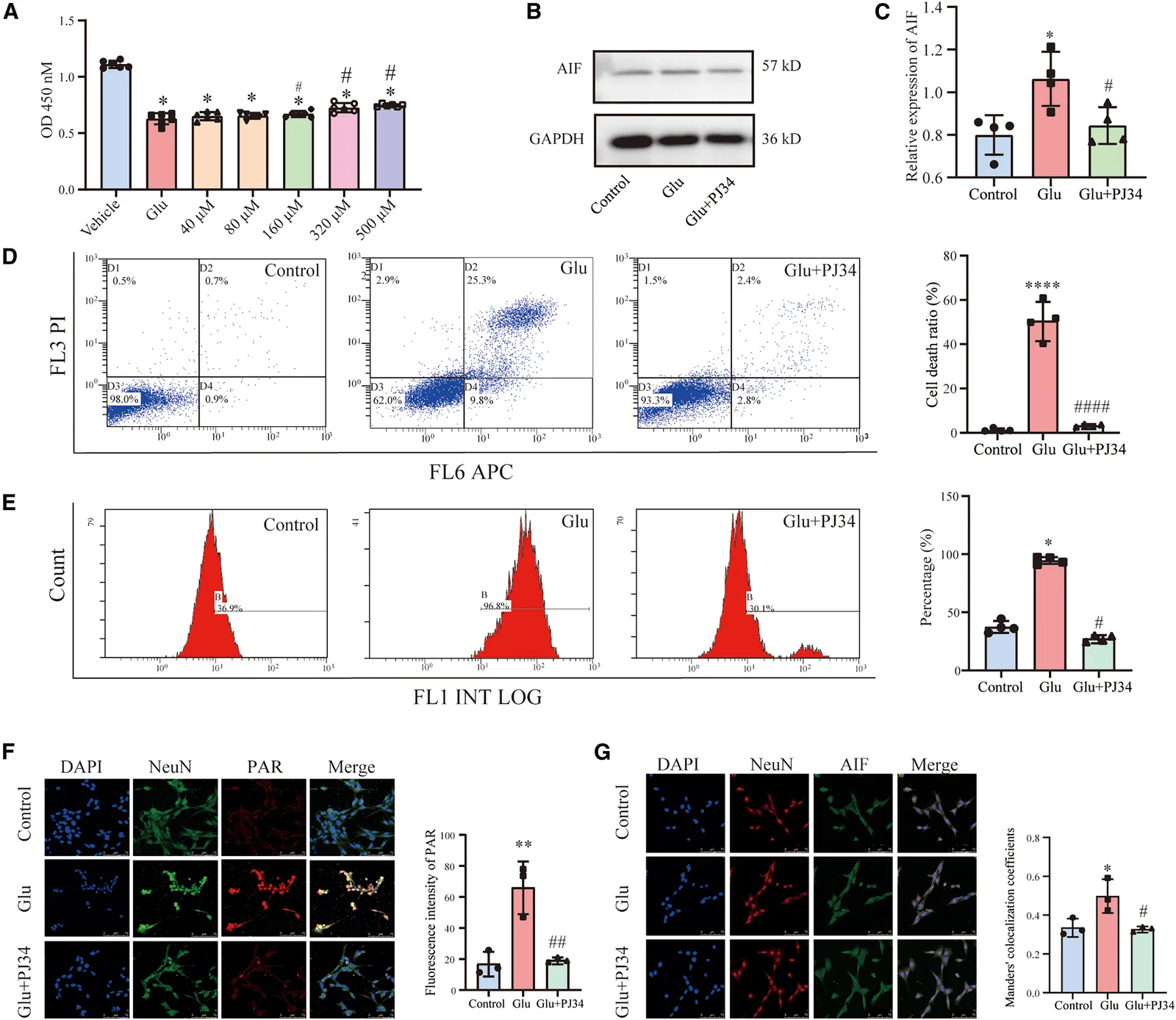
Administration of PJ34 relieved parthanatos in HT22 cells induced by glutamate (A) Viability of HT22 cells measured by CCK8 assay (*n* = 5, independent repeats). (B and C) Expression of AIF determined by western blotting (*n* = 4, independent repeats). (D) Cell death ratio of HT22 cells reflected by flow cytometric analysis (*n* = 4, independent repeats). (E) ROS production in HT22 cells reflected by flow cytometric analysis (*n* = 4, independent repeats). (F and G) Expression of PAR and localization of AIF within the nuclei (*n* = 3, independent repeats). The data are expressed as mean ± SD and analyzed for statistical significance using one-way ANOVA (∗*p* < 0.05, ∗∗*p* < 0.01, ∗∗∗∗*p* < 0.0001 versus control group; #*p* < 0.05, ##*p* < 0.01, ####*p* < 0.0001 versus Glu group). Scale bars, 25 μm.

### miR-7a-5p overexpression attenuated parthanatos in HT22 cells

In order to clarify the effect of miR-7a-5p on parthanatos, we generated a miR-7a-5p overexpressing cell line through lentivirus transfection in HT22 cells (Figure 7A). We observed that L-glutamate significantly reduced the cell viability of HT22 cells, which was reversed by miR-7a-5p overexpression (Figure 7B). Meanwhile, miR-7a-5p could reduce the cell death ratio induced by L-glutamate (Figure 7C). Mitochondria play roles in the process of parthanatos, and we found that miR-7a-5p overexpression can prevent the MMP loss and reduce the ATP consumption induced by L-glutamate (Figures 7D and S3). Both PARP1 and AIF expression abundance were reduced by miR-7a-5p overexpression (Figure 7E). Moreover, miR-7a-5p overexpression can reduce PAR formation and AIF Mander’s coefficients (Figures 7F and 7G).

**Figure 7:**
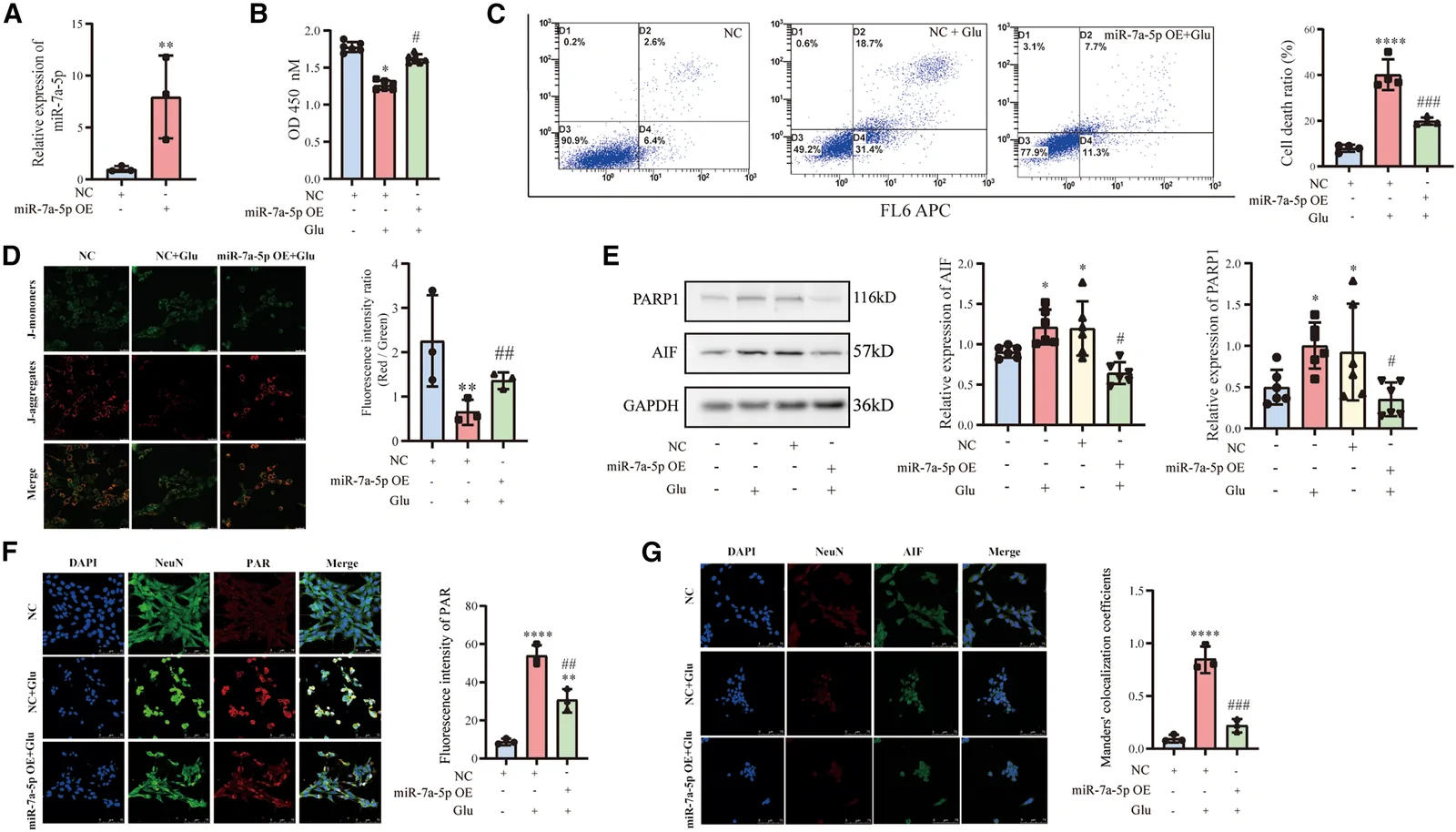
Overexpression of miR-7a-5p alleviated parthanatos in HT22 cells induced by glutamate (A) Validation of lentivirus-mediated miR-7a-5p overexpression by real-time PCR (*n* = 3, independent repeats). (B) HT22 cells viability measured by CCK8 assay (*n* = 6, independent repeats). (C) Cell death ratio of HT22 cells detected by flow cytometric analysis (*n* = 4, independent repeats). (D) Mitochondrial membrane potentials in HT22 cells determined by JC-1 staining (*n* = 3, independent repeats). (E) Western blotting analysis showed the expression levels of PARP1 and AIF (*n* = 6, independent repeats). (F and G) Expression of PAR and nuclear localization of AIF (*n* = 3, independent repeats). The data are expressed as mean ± SD and analyzed for statistical significance using one-way ANOVA (∗*p* < 0.05, ∗∗*p* < 0.01, ∗∗∗∗*p* < 0.0001 versus control group, #*p* < 0.05, ##*p* < 0.01, ###*p* < 0.001 versus Glu group), Scale bars, 25 μm.

### miR-7a-5p attenuated parthanatos in HT22 cells in a PARP1-dependent manner

To further determine whether PARP1 is the indispensable downstream molecule when miR-7a-5p exerts its parthanatos-suppressing effect, we detected the impact of simultaneous activation of miR-7a-5p and PARP1. Cells were exposed to different concentrations of PARP1 agonist, N-methyl-N′-nitro-nitrosoguanidine (MNNG), prior to L-glutamate incubation. And we observed a 12 h incubation with MNNG (5 μM) significantly reversed the protective effect of miR-7a-5p overexpression on cell viability (Figure 8A). Moreover, a 12-h incubation with MNNG (1 and 5 μM) effectively reversed the protective effect of miR-7a-5p overexpression against cell cytotoxicity (Figure 8B). These results also suggest that the miR-7a-5p/PARP1 pathway is indispensable for regulating L-glutamate-induced parthanatos in HT22 cells.

**Figure 8:**
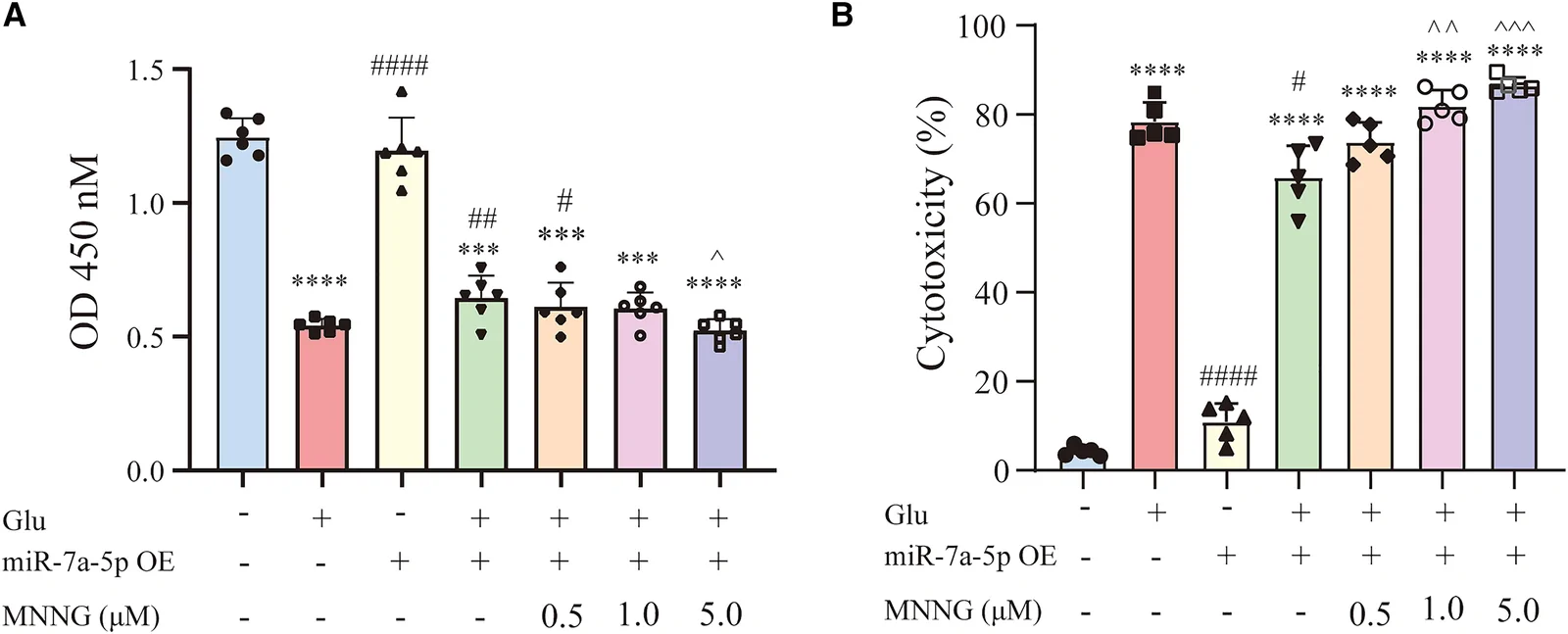
PARP1 agonist MNNG diminished the neuroprotective effect of miR-7a-5p (A) HT22 cells viability measured by CCK8 (*n* = 6, independent repeats). (B) Cytotoxicity of HT22 cells was measured by LDH assay (*n* = 5, independent repeats). The data are expressed as mean ± SD and analyzed for statistical significance using one-way ANOVA (∗∗∗*p* < 0.001, ∗∗∗∗*p* < 0.0001 versus control group; #*p* < 0.05, ##*p* < 0.01, ####*p* < 0.001 versus Glu group; ^∧^*p* < 0.05, ^∧∧^*p* < 0.01, ^∧∧∧^*p* < 0.001 versus miR-7a-5p OE + Glu group).

## Discussion

HIE remains a leading cause of neonatal mortality and neurodevelopmental disability. Up to now, only hypothermia has been used as an effective treatment for neonatal HIE in clinical practice.^19^ However, approximately 50% of infants who receive hypothermic treatment still die or suffer from permanent neurobehavioral and cognitive dysfunction.^20^ Here, we identify parthanatos—a PARP1-dependent form of programmed necrosis—as a central pathological mechanism in HIE and demonstrate that miR-7a-5p suppresses neuronal death by targeting PARP1, offering a potential therapeutic strategy.

Despite being enriched in neurons, miR-7a-5p is not exclusively expressed in these cells. Accumulating evidence indicates that miR-7a-5p also exerts functional roles in glial cells. For instance, miR-7-mediated suppression of NLRP3 in microglia/macrophages reduces inflammatory cytokine production and promotes neuroprotection following intracerebral hemorrhage in rats.^21^ In astrocytes, miR-7 targets endoplasmic reticulum (ER) stress to attenuate inflammatory responses and cell damage under oxygen-glucose deprivation conditions.^22^ Moreover, HIV-1 trans-activator of transcription (Tat) protein induces astrocyte-derived extracellular vesicles enriched with miR-7, which, upon neuronal uptake, downregulate NLGN2 and lead to synaptic alterations.^23^ These findings suggest that glial cells can regulate neuroinflammation and even modulate neuronal function through miR-7a-5p. Nevertheless, the developing brain has extremely high energy demands, and post-mitotic neurons exhibit inherent metabolic and functional vulnerability. Consequently, energy depletion, excitotoxicity, oxidative stress, and parthanatos converge most severely on neurons. Furthermore, the clinical sequelae of HIE, including motor and cognitive disabilities, directly reflect neuronal loss in specific brain regions. Therefore, protecting neurons from injury is a critical therapeutic goal in HIE.

Our study robustly establishes parthanatos as the dominant executor of neuronal death in our experimental HIE model. *In vivo*, the observed suite of motor deficits—prolonged righting reflex, impaired performance on the inclined plane and balance beam tests—provides a functional correlate to the cellular catastrophe. Ultrastructural evidence of mitochondrial cristae disruption points to profound bioenergetic failure, a hallmark of this pathway. Crucially, the biochemical signature we observed is definitive: there is a significant accumulation of PAR polymer and the translocation of AIF from mitochondria to the nucleus, while classical apoptosis markers (cleaved PARP1 and activated caspase-3) remained absent. This pattern is particularly significant in the context of the neonatal brain. The immature brain exhibits a distinct excitotoxic profile with heightened sensitivity to glutamate, attributed to the incompleteness of both the glutamate clearance system and the antioxidant defense system. This primes the system for the massive DNA damage that triggers PARP1 hyperactivation. Unlike apoptosis, which is an energy-dependent, orderly process, parthanatos is a metabolically explosive event. Hyperactive PARP1 consumes NAD+ at an unsustainable rate, leading to ATP depletion, loss of mitochondrial membrane potential (MMP), and rampant production of ROS. The subsequent release and nuclear translocation of AIF, a flavoprotein possessing DNase activity, facilitates extensive DNA fragmentation and chromatin condensation, ultimately leading to rapid necrotic cell death.

Although the pathophysiology of HIE involves multiple factors, including excitotoxicity, energy failure, oxidative stress, acidosis, and neuroinflammation, oxidative stress serves as a central hub that converges and amplifies downstream injury. The glutamate-induced HT22 cell injury model is a well-established and effective tool for studying oxidative stress-induced neuronal death in HIE. In HT22 cells, glutamate inhibits the cystine/glutamate antiporter (system Xc^−^), leading to glutathione depletion and robust ROS production. This simplified system allows us to focus on the miR-7a-5p/PARP1/parthanatos axis in a controlled context, isolating the interference of additional confounding factors present *in vivo*. It should be noted that, under this model, PARP1 overactivation still leads to energy depletion (ATP decline) as a downstream event of parthanatos, which is consistent with the *in vivo* pathophysiology. The recapitulation of this entire cascade, based on the oxidative stress *in vitro* model of glutamate-exposed HT22 neurons and using specific inhibition by PJ34, confirms the central mechanistic role of PARP1 in driving this injury phenotype. Our work thus extends the paradigm of parthanatos from adult stroke models to the unique developmental and metabolic landscape of perinatal HIE, suggesting it may be a particularly vulnerable node in the neonatal brain’s response to severe hypoxia-ischemia.

Given the destructive potential of unrestrained parthanatos, endogenous regulatory mechanisms must exist. Expanding evidence has prompted investigation into the potential use of miRNAs in disease therapy, and several approaches for miRNA delivery have entered clinical trial.^24^ Building on bioinformatics analysis of HIE patient data, we identified miR-7a-5p as a key endogenous suppressor of parthanatos in HIE. MiR-7a-5p has been reported to be highly expressed in the mammalian brain and participate in the development of the cerebral cortex cells.^25^^,^^26^^,^^27^ Another study reported that miR-7a-5p mitigated ischemic brain damage by repressing α-synuclein and was necessary for functional recovery after stroke.^28^

While its reported roles in cortical development and post-stroke recovery have hinted at neuroprotective functions, our study defines a precise and powerful mechanism: the direct post-transcriptional repression of PARP1. The downregulation of miR-7a-5p observed in HIE rats, both in the cortex and peripherally, may represent an endogenous defense response, albeit insufficient. Moreover, we validated that miR-7a-5p directly binds to the 3′ UTR of PARP1 mRNA,^29^ and its downregulation (as seen in specific cellular contexts of our model) correlates with pathological PARP1 protein elevation. Functionally, restoring miR-7a-5p levels was sufficient to rescue neuronal viability, restore mitochondrial integrity, and suppress the key hallmarks of parthanatos—PAR polymer formation and AIF translocation. Studies have revealed that PARP1 knockout protected animals from hypoxic-ischemic injury, lipopolysaccharide (LPS)-induced shock, and diet-induced obesity.^30^^,^^31^ The miR-7a-5p directly binds the 3′ UTR of PARP1; its downregulation in HIE rats and glutamate-treated neurons correlated with PARP1 upregulation and parthanatos activation. Overexpression of miR-7a-5p rescued neuronal viability, restored mitochondrial function, and suppressed PAR polymer formation and AIF nuclear translocation. The converse experiment, where PARP1 was pharmacologically activated using MNNG, completely abolished the protective effect of miR-7a-5p overexpression, cementing the linear causality within this axis. These results expand the known neuroprotective roles of miR-7a-5p in stroke and myocardial ischemia to perinatal HIE, highlighting its therapeutic potential. By tonically inhibiting PARP1 expression, miR-7a-5p may help raise the threshold for PARP1 hyperactivation, potentially keeping excitotoxic or oxidative insults from triggering the catastrophic feedforward cycle of energy depletion and necrosis.

The elucidation of the miR-7a-5p/PARP1 axis holds promise for future therapeutic development, providing a rationale for the creation of adjuvant therapies to complement hypothermia. While hypothermia modulates multiple injury processes (e.g., metabolic rate, excitotoxicity, and inflammation), it may not directly or sufficiently inhibit the vigorous parthanatos pathway, once initiated. Strategies to boost miR-7a-5p activity—such as synthetic miR-7a-5p mimics or agents that enhance its endogenous expression—could directly target this deficiency. This approach holds potential advantages over direct PARP1 pharmacological inhibitors, as it may offer a more nuanced, physiological form of regulation that modulates pathological overexpression while sparing the essential DNA repair functions of basal PARP1 activity. However, significant translational challenges must be navigated. These include optimizing the delivery of miRNA-based therapeutics across the neonatal blood-brain barrier, defining the critical therapeutic window beyond the immediate perinatal period, and rigorously assessing long-term safety in developing organisms. Furthermore, the persistent elevation of miR-7a-5p in the peripheral blood of HIE rats suggests its potential utility as a prognostic or pharmacodynamic biomarker, a possibility worthy of future clinical correlation studies. Future research should also investigate the potential synergistic effects of combining miR-7a-5p augmentation with hypothermia and explore whether this axis is similarly relevant in models of diffuse versus focal brain injury in neonates.

Our findings establish parthanatos as a critical driver of neuronal death in HIE and identify the miR-7a-5p/PARP1 axis as a novel therapeutic target. By attenuating parthanatos, miR-7a-5p could complement existing therapies, offering hope for improving outcomes in this neonatal condition. Our study demonstrates that neonatal hypoxic-ischemic injury-induced motor and cognitive deficits are primarily driven by PARP1-mediated parthanatos. This dominant cell death pathway is suppressed by miR-7a-5p through direct targeting of PARP1, positioning miR-7a-5p as a promising therapeutic candidate for alleviating both neurological and cognitive impairments in HIE.

### Limitations of the study

While we examined the overall neuroprotective effect of miR-7a-5p against glutamate-induced HT22 cell injury and cerebral damage, there are several limitations to our study. First, HIE involves energy failure (ATP/NAD^+^ depletion), acidosis, neuroinflammation, and blood-brain barrier disruption. The glutamate-induced HT22 cell injury model cannot replicate these combined insults. Second, while our study primarily investigates the role of the miR-7a-5p/PARP1 axis in neuronal parthanatos, it is imperative to further explore whether this axis also operates within glial cells and contributes to the neuroinflammatory aspects of HIE damage. Future studies should utilize more sophisticated co-culture systems or animal models with pharmacological interventions to elucidate the specific role of this pathway within the complex environment of HIE. Third, this study lacks imaging data. Functional magnetic resonance imaging (fMRI) will be introduced in future work to provide additional evidence at both macroscopic structural and functional metabolic levels.

## Resource availability

### Lead contact

Requests for additional information, resources, or reagents should be addressed to the lead contact, Bing Li (libingbm@163.com).

### Materials availability

No new reagents were generated in this study.

### Data and code availability

• This study analyzes existing, publicly available data. The source for the dataset is listed in the key resources table (GEO: GSE121178).
• This study does not report the original code. The R package was accessed through an online tool (https://www.xiantao.love), which was used to analyze the data and produce plots.
• Any additional information required to reanalyze the data used in this study is available from the lead contact upon request.

## Acknowledgments

Electron microscope analysis of this work was supported by Hongyang Gao, Electron Microscopy Core Laboratory, School of Basic Medical Sciences, and 10.13039/501100003347Fudan University.

This research was funded by the 10.13039/501100001809National Natural Science Foundation of China (grant numbers 82305428, 82174522, and 81774444), Jinshan Hospital Affiliated to Fudan University Youth Scientific Research Initiation Fund (grant number JYQN-JC-202401), Pediatrics-the Medical Key Disciplines of Shanghai (grant number 2024ZDXK0056) and Clinical Integration of Traditional Chinese and Western Medicine, and Shanghai Municipal Health System Key Discipline (grant number 2024ZDXK0025).

## Author contributions

D.C. and X.K. supervised the experiments and analyzed the data; H.Z., Y.Z., R.Q., C.S., A.K., and Y.S. conducted the experiments and recorded the data; B.L. and D.C. designed the study and drafted the manuscript.

## Declaration of interests

The authors declare no conflicts of interest.

## STAR★Methods

### Key resources table

**Table:** 

REAGENT or RESOURCE	SOURCE	IDENTIFIER
**Antibodies**		

PARP1	Cell Signaling Technology	Cat# 9542; RRID: AB_2160739
Caspase3	Cell Signaling Technology	Cat# 9662; RRID: AB_331439
AIF	Bioss Antibodies	Cat# bs-0037R; RRID: AB_10856378
MIF	Cell Signaling Technology	Cat# 88186S; RRID: AB_2800118
GPX4	Cell Signaling Technology	Cat# 59735; RRID: AB_2940796
ACSL4	ABclonal	Cat# A20414; RRID: AB_2909505
RIP3	Cell Signaling Technology	Cat# 15828; RRID: AB_2722663
p-RIP3	Affinity	Cat# AF7443; RRID: AB_2843883
MLKL	Affinity	Cat# DF7412; RRID: AB_2839350
*p*-MLKL	Affinity	Cat# AF7420; RRID: AB_2843860
β-Actin	EpiZyme	Cat# LF212; RRID: AB_3678780
GAPDH	Cell Signaling Technology	Cat# 5174; RRID: AB_10622025
PAR	Merck	Cat# AM80; RRID: AB_2155072

**Chemicals, peptides, and recombinant proteins**		

DMEM	Gibco	C11965500BT
BSA	Gibco	10091148
Triton X-100	Sigma-aldrich	SLBT3016
PBS	Gibco	70011044
TWEEN 20	Merck	P2287-500ML
PJ34	Proteintech	CM11523
MNNG	Abmole	M9797-5g

**Critical commercial assays**		

Cell Counting Kit-8 (CCK-8) assay	Dojindo	CK13-500tests
Annexin V, 633 Apoptosis Detection Kit	Dojindo	AD11-50assays
Reactive Oxygen Species Assay Kit	Beyotime	S1105M
Mitochondrial Membrane Potential Assay Kit	Beyotime	C2006
ATP assay	Solarbio	BC0305
RNA-Quick Purification Kit	Yishan Biotechnology	RN001
TB Green Premix Ex Taq (Tli RNaseH Plus)	Takara	RR420B
Dual-Glo® Luciferase Assay System	Promega	E2920

**Deposited data**		

Raw and analyzed data	This paper	GEO: GSE121178 (https://www.ncbi.nlm.nih.gov/geo)
Online R-based tool for data analysis and plotting	This paper	https://www.xiantao.love

**Experimental models: Cell lines**		

HT22	FuHeng Cell Center	N/A
HEK293T	Beyotime	N/A

**Experimental models: Organisms/strains**		

Sprague-Dawley rats	Shanghai Slac Laboratory Animal Co. Ltd	N/A

**Oligonucleotides**		

miR-7a-5p primer (forward 5′-ACCGGTGGAAGACTAGTGATTTTGTTGT-3′).	This paper	N/A
U6 primer (forward 5′-GGAACGATACAGAGAAGATTAGC-3′, reverse 5′-TGGAACGCTTCACGAATTTGCG-3′).	This paper	N/A

**Software and algorithms**		

SPSS version 22	N/A	IBM, USA
NCBI Gene Expression Omnibus database	N/A	https://www.ncbi.nlm.nih.gov/geo
R v4.2.1 software	N/A	https://www.r-project.org

### Experimental model and study participant details

#### Institutional review board statement

The study was approved by the Animal Welfare & Ethics Committee of Shanghai Public Health Clinical Laboratory (Shanghai, China) and internationally accredited guidelines and ethical regulations (approval number NO.2022-A004-01). All animals were cared for in accordance with internationally accredited guidelines.

#### Animal

Pregnant female Sprague-Dawley rats were procured from the Slac Laboratory Animal Company (Shanghai, China). Postpartum, the offspring were nursed by their dams until the weaning stage. Male offspring, aged seven days, were selected for subsequent experimental procedures. All animals were randomly assigned to groups using a standardized protocol and housed in a specific pathogen-free (SPF) facility at the Shanghai Public Health Clinical Center. They were maintained under standard laboratory conditions (22°C ± 1°C,50% ± 10% humidity, 12-h light/dark cycle) with *ad libitum* access to food and water.

#### Cell culture

HT22 cells (FuHeng Cell Center, Shanghai, China) were cultured in DMEM/10% FBS (Gibco/Thermo Fisher Scientific, Waltham, MA, USA) under condition of 37°C and 5% CO_2_. The cell lines used in this study were authenticated by Short Tandem Repeat (STR) profiling to confirm their identity and genetic consistency. And the cells were regularly tested for mycoplasma contamination and found to be negative.

HEK293T cells (C6008, Beyotime, Shanghai, China) were cultured in DMEM/10% FBS under condition of 37°C and 5% CO_2_. The cell lines used in this study were authenticated by Short Tandem Repeat (STR) profiling to confirm their identity and genetic consistency. And the cells were regularly tested for mycoplasma contamination and found to be negative.

### Method details

#### Data acquisition, processing, and differential miRNA expression analysis

The HIE datasets GSE121178 from GPL22120 were downloaded from the NCBI Gene Expression Omnibus database by “GEO query” package of R v4.2.1 software, including 3 HIE samples and 3 non-HIE human whole blood samples. Data were normalized again through the normalizeBetweenArrays function of the “limma” package. A boxplot was generated to visualize the effect of removing inter-sample differences, and the effect of inter-sample correction was assessed using a two-dimensional principal component analysis (PCA) cluster plot. Differentially expressed miRNAs were screened with the “limma” package. A volcano map was constructed using the “ggplot2” package to visualize the differentially expressed miRNAs with a fold change of >1.0 and *p*-value <0.05.

#### Animal model

Male Sprague-Dawley rat pups underwent hypoxic ischemic brain injury on P7 via unilateral carotid artery ligation followed by 8% O_2_ exposure for 3.5 h at 37°C.^32^ The 3.5 h hypoxia duration, longer than the typical 1.5–2 h in the Rice Vannucci model, was empirically established under our laboratory conditions. Pilot experiments showed that shorter durations (1.5–2.5 h) yielded inconsistent infarct volumes, whereas 3.5 h reliably produced a stable unilateral infarction without excessive mortality, enabling robust assessment of neuroprotective efficacy. Sham controls received artery exposure without ligation/hypoxia. Brain infarct size was evaluated using 2,3,5-Triphenyltetrazolium chloride (TTC) staining.

#### Intracerebroventricular injection

The miR-7a-5p agomir was administered via intracerebroventricular injection at 6 h post-hypoxia-ischemia (HI). Under isoflurane anesthesia, a midline scalp incision was made to expose the skull. The injection coordinates were 1.0 mm posterior and 1.0 mm lateral to bregma, with a depth of 2.5 mm. A total of 10 nmol of miR-7a-5p agomir or negative control agomir (agomir-NC) dissolved in 5 μL of sterile 0.9% NaCl was slowly injected into the lateral ventricle using a microsyringe. Sham-operated animals received the same volume (5 μL) of 0.9% NaCl injected into the lateral ventricle.

#### Behavior tests

Righting reflex of neonatal rats was assessed from P7 to P11, according to the literature and previous studies.^33^^,^^34^ During this test, the rat pups were placed in a supine position on a cleaned flat surface and the time for these pups to turn over to the prone position with all the four paws was recorded.

Inclined plane test was used to measure limb strength on the day of P14, as described previously.^35^ The rat pups were placed on the plane and the angle of the plane was increased by 5° every 5 s, recording the max angle of the slope to which the pups could still hold.

Balance beam test was performed to assess motor coordination on P34 to observe long-term outcome of HIE.^36^ First, the rats were trained for 3 consecutive days. When test, the rats were placed at the end of the beam and they would move toward the other end with a home box. During the final test, time to cross the balance beam and the number of slips of the hind limbs were recorded.

Long term cognitive function was assessed at P30 using the novel object recognition (NOR) test. The test was conducted over two consecutive days. On day 1 (habituation), each rat was placed in an empty arena for 5 min. On day 2, the rat first explored two identical objects (A1 and A2) for 5 min (familiarization phase). After a 1-h interval, object A2 was replaced with a novel object (B), and the rat was returned to the arena for another 5 min (test phase). Memory performance was assessed using the discrimination index calculated as follows: Discrimination index = T_B_/(T_A_ + T_B_) ∗100%, where T_A_ is the time spent exploring the familiar object and T_B_ is the time spent exploring the novel object.

#### Cell treatment and lentiviral transduction

HT22 cells were incubated with 5 mM L-glutamate for 12 h to induce oxidative cell injury. For parthanatos inhibition, cells were pretreated with 320 μM PJ34 (PARP1 inhibitor, APExBIO, USA) for 1 h prior to glutamate exposure. Parthanatos was induced by 0.5 μM, 1 μM and 5 μM MNNG (Merck Millipore, USA) in HT22 cells for 1 h prior to glutamate exposure. For miR-7a-5p overexpression, cells (5×10^3^ cells/10 cm dish) were transduced with lentivirus (Obio Technology, Shanghai, China) carrying pGV514-miR-7a-5p or empty vector (MOI = 20) at 30–45% confluence. After 48 h, transduction efficiency was confirmed by fluorescence microscopy, and miR-7a-5p expression was validated via qPCR.

#### Electron microscope

Dissected brains were fixed with 2.5% glutaraldehyde for 2 h, dehydrated, and embedded in Epon resin. Ultra-thin sections (80 nm) were stained with uranyl acetate and lead citrate, then imaged using a transmission electron microscope (Philips CM120, Eindhoven, The Netherlands).

#### Cellular functional assays

Cell viability: HT22 cells (1×10^4^/well) were treated with glutamate, and viability was assessed using the Cell Counting Kit-8 (CCK-8) assay (Dojindo Molecular Technologies, Kumamoto, Japan) according to the manufacturer’s instruction. 10 μL CCK8 solution was added into each well. After incubated in 37°C for 1h, absorbance at 450 nm was measured using a Multiscan MS spectrophotometer (Labsystems, Stockholm, Sweden).

Apoptosis: Cells were stained with Annexin V-633 and propidium iodide (PI) (Dojindo Molecular Technologies, Kumamoto, Japan), then analyzed by flow cytometry (Beckman Coulter, USA).

ROS levels: Cells were loaded with the fluorescent probe DCFH-DA from Beyotime (Haimen, China) for 20 min, and fluorescence intensity was quantified via flow cytometry.

Mitochondrial membrane potential: Glutamate-treated cells were stained with JC-1 dye and imaged by fluorescence microscopy (Olympus, Japan).

ATP assay:Cells were collected for ATP extraction following the assay kit protocol (Solarbio, Beijing, China). Extraction was conducted under ice-cold condition using extractive solution and trichloromethane.

Then, samples were incubated with working solution. Absorbance at 340 nm was measured using a Multiscan MS spectrophotometer (Labsystems, Stockholm, Sweden).

#### Real-time PCR

Total RNA was extracted from tissue or cells using RNA-Quick Purification Kit (Yishan, Shanghai, China). The real-time PCR was performed with the 7500 real-time PCR system (Applied Biosystems, USA). miR-7a-5p expression was quantified with TB Green reagents (Takara, Japan) and normalized to U6. The primer sequences were listed as follows: miR-7a-5p primer (forward 5′-ACCGGTGGAAGACTAGTGATTTTGTTGT-3′), U6 primer (forward 5′-GGAACGATACAGAGAAGATTAGC-3′, reverse 5′-TGGAACGCTTCACGAATTTGCG-3′).

#### Luciferase reporter assay

TargetScan (www.targetscan.org) was used to predict the association between miR-7a-5p and PARP1. The wild-type (WT) PARP1 3′-UTR containing the binding site for miR-7a-5p or mutated (MUT) PARP1 3′-UTR was cloned into pGL4 Luciferase Reporter Vectors. Then, the vector was transfected into HEK293T cells (FuHeng Cell Center, Shanghai, China) with miR-7a-5p mimic or negative control (NC) using Lipofectamine 2000 (Invitrogen, USA). Luciferase activity was assessed using Dual-Glo Luciferase Assay System (Promega, USA).

#### Protein expression and localization analysis

##### Western blotting

Protein lysates from cortical tissues or HT22 cells were extracted using SDS lysis buffer. Proteins (20 μg) were separated by 7.5% SDS-PAGE, transferred to PVDF membranes (Millipore, USA), and probed with antibodies against PARP1 (1:1000, 9542, Cell Signaling Technology), Caspase3 (1:1000, 9662, Cell Signaling Technology), AIF (1:1000, bs-0037R, Bioss Antibodies), MIF (1:1000, 88186S, Cell Signaling Technology), GPX4 (1:1000, E5Y8K, Cell Signaling Technology), ACSL4 (1:1000, A20414, ABclonal), RIP3 (1:1000, D8J3L, Cell Signaling Technology), p-RIP3 (1:1000, AF7443, Affinity), MLKL (1:1000, DF7412, Affinity), *p*-MLKL (1:1000, AF7420, Affinity),β-Actin (1:1000, LF212, EpiZyme, China) and GAPDH (1:1000, 5174, Cell Signaling Technology). HRP-conjugated secondary antibodies and ECL detection were used for visualization.

##### Immunofluorescence

Formalin-fixed brain sections were embedded in paraffin and sliced (4 μm). Sections were incubated with anti-PAR (1:100, AM80, Merck) and anti-AIF (1:500, bs-0037R, Bioss Antibodies) antibodies, followed by Alexa Fluor 594/488-conjugated secondary antibodies. Nuclei were counterstained with DAPI (0100-20, Southern Biotech). Images were captured using a confocal microscope (Leica sp5, Germany).

#### Statistical analysis

All experimental data were expressed as the mean ± SD. SPSS 22.0 software was used for statistical analysis. Student’s *t* test (parametric) was used to perform comparisons of two groups, and one-way analysis of variance followed by Tukey’s post hoc test was used to compare multiple groups (*p* < 0.05 were considered statistically significant).

## Supplemental information

Document S1. Figures S1–S3 and Table S1

Table S2. Association of microRNAs with diseases

Data S1. Uncropped western blot images for all figures
