# Supplementary material for: miR-7a-5p attenuates hypoxic-ischemic encephalopathy by suppressing PARP1-mediated parthanatos
Source: iScience. 2026 Oct 27;29(11):117947. doi: 10.1016/j.isci.2026.117947 (PMC13734944; doi:10.1016/j.isci.2026.117947)
Supplement: Supplementary file 1 [file mmc1.pdf]

iScience, Volume *29*

Supplemental information

miR-7a-5p attenuates hypoxic-ischemic encephalopathy by suppressing PARP1-mediated parthanatos

Danmei Chen, Xiangyu Kong, Hequan Zhong, Yunpeng Zhang, Chenqin Si, Ayipaxiaguli Kasimu, Rui Qiao, Yuting Song, and Bing Li

Supplementary Materials

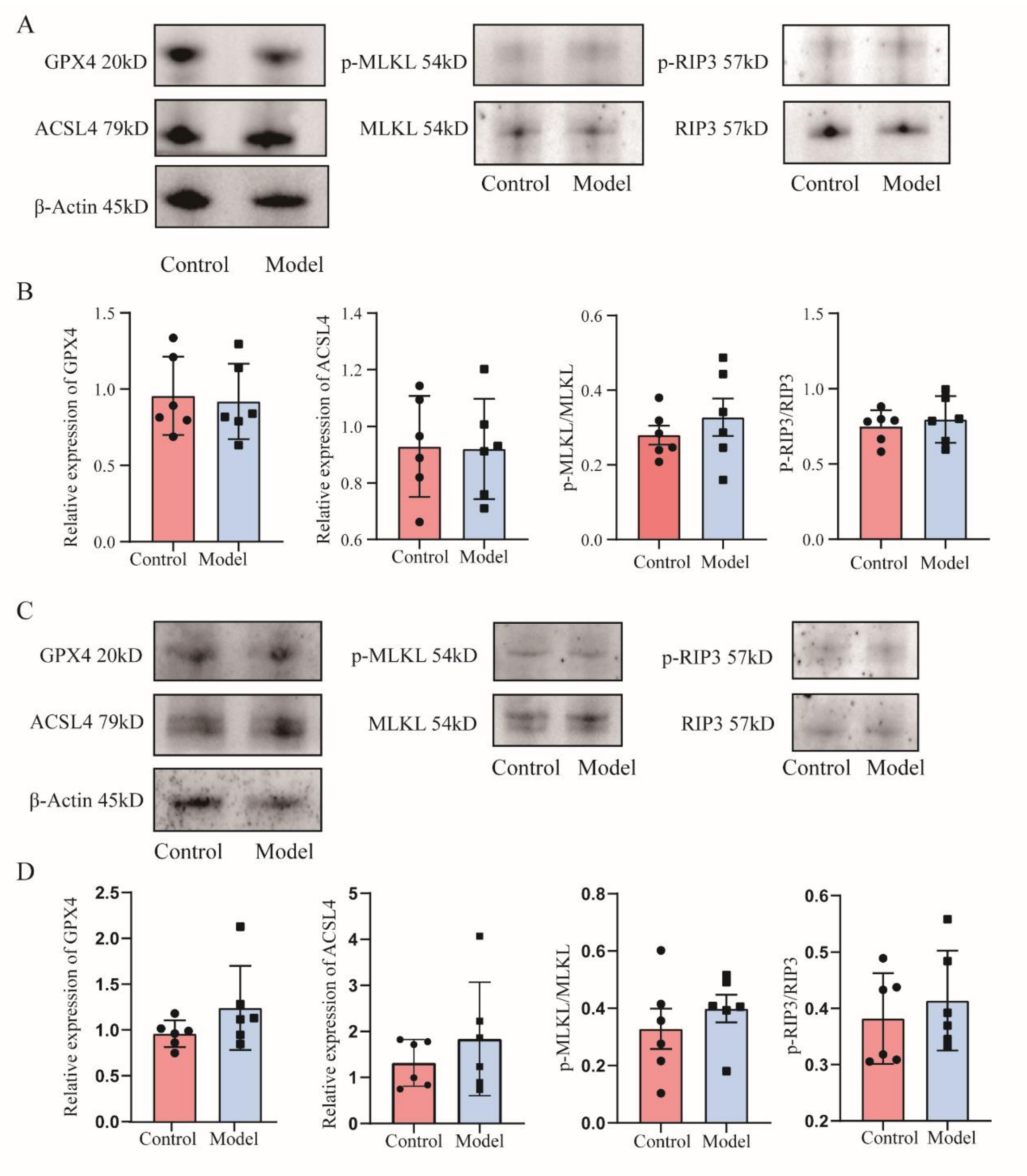

Figure S1. Expression of ferroptosis and necroptosis-related proteins in glutamate-induced neuronal injury model and rat HIE model. (A, B) Western blot analysis of GPX4, ACSL4, p-MLKL, MLKL, p-RIP3 and RIP3 protein levels in the glutamate-induced neuronal injury model (n = 6 separate culture replicates). (C, D) Western blot analysis of GPX4, ACSL4, p-MLKL, MLKL, p-RIP3 and RIP3 protein levels in the rat HIE model (n = 6 individual animals per group). The data are expressed as mean ± SD and analyzed for statistical significance using t-test.

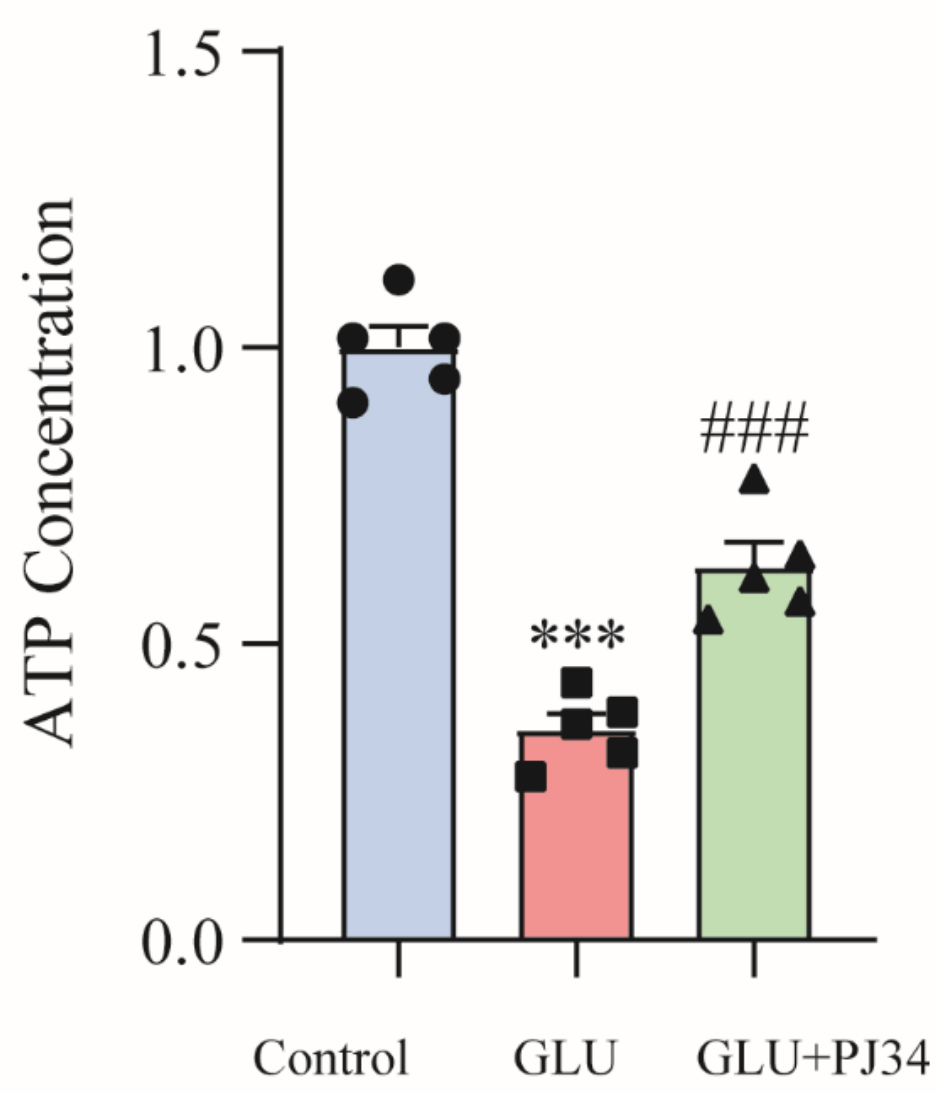

Figure S2. Relative cellular ATP production in glutamate-induced HT22 with or without PJ34 pretreatment. The data are expressed as mean ± SD and analyzed for statistical significance using one-way ANOVA (***p < 0.001 versus control group, ###p < 0.001 versus Glu group, n = 5 separate culture replicates).

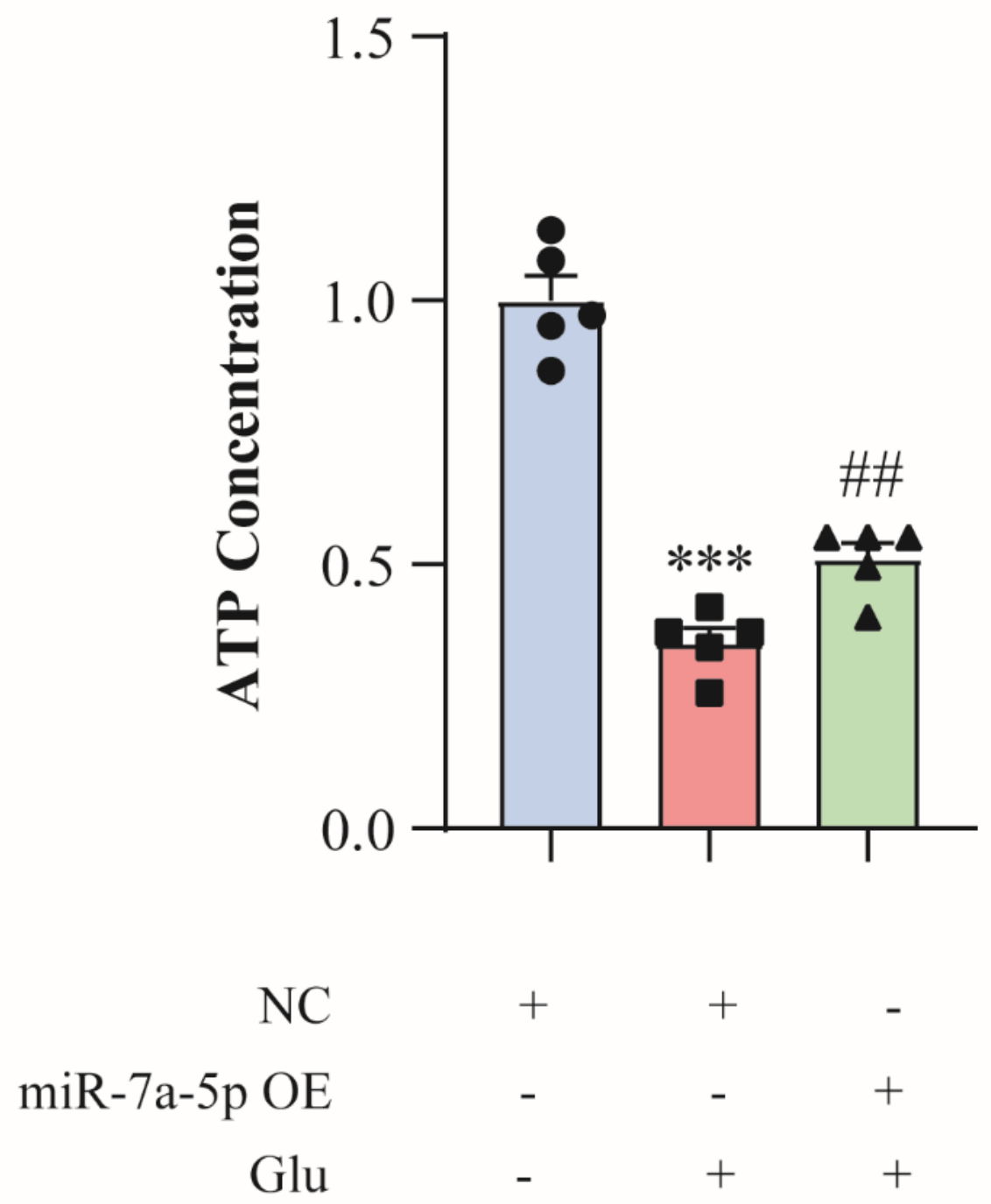

Figure S3. Relative cellular ATP production in glutamate-induced HT22 with NC or miR-7a-5p overexpression vector pretreatment. The data are expressed as mean ± SD and analyzed for statistical significance using one-way ANOVA (***p < 0.001 versus control group, ##p < 0.01 versus Glu group, n = 5 separate culture replicates).

Table S1. Differential expressed microRNAs in the hypoxic-ischemic encephalopathy blood samples compared with normal blood samples

Symbol	logFC	P.Value
MIR4252	2.554705	0.015844
MIR320E	-2.2142	0.045541
MIR6869	1.949656	0.034569
MIR1827	1.928844	0.004152
MIR329-2	1.787073	0.009624
MIR3156-1	1.782878	0.000631
MIR27B	1.769937	0.006772
MIR8083	1.690338	0.04465
MIR4508	-1.61248	0.021256
MIR7112	1.586811	0.024233
MIR3922	1.572533	0.010803
MIR874	1.561386	0.011874
MIR30B	1.546137	0.036625
MIR4730	1.542251	0.013778
MIR6847	1.532482	0.004684
MIR125B2	1.524135	0.001596
MIR3150A	1.469471	9.2E-05
MIR30D	-1.46912	0.000477
MIR4526	1.448166	0.028412
MIR3169	1.427263	0.006342
MIR490	-1.41825	0.012366
MIR4701	-1.34109	0.046003
MIR4527	1.338117	0.01396
MIR3131	1.325218	0.016777
MIR182	1.310188	0.019564
MIR527	-1.30157	0.006917
MIR4458	-1.28986	0.016166
MIR4263	-1.26911	0.005107
MIR3978	1.227952	0.019361
MIR665	1.219772	0.011091
MIR1272	1.209199	0.018357
MIR4708	1.182042	0.035646
MIR146B	1.175284	0.033883
MIR6765	1.143664	0.036867
MIR4768	-1.1338	0.005949
MIR4435-1	1.114202	0.039518
MIR4745	1.107699	0.019101
MIR6134	1.032611	0.001353
MIR7-2	-1.00779	0.005795
MIR7156	1.002728	0.010489
