# Supplementary material for: miR-7a-5p attenuates hypoxic-ischemic encephalopathy by suppressing PARP1-mediated parthanatos
Source: iScience. 2026 Oct 27;29(11):117947. doi: 10.1016/j.isci.2026.117947 (PMC13734944; doi:10.1016/j.isci.2026.117947)

Fig 3E

AIF

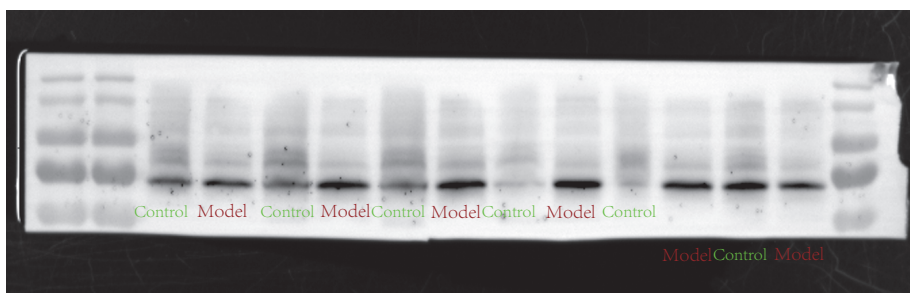

PAR

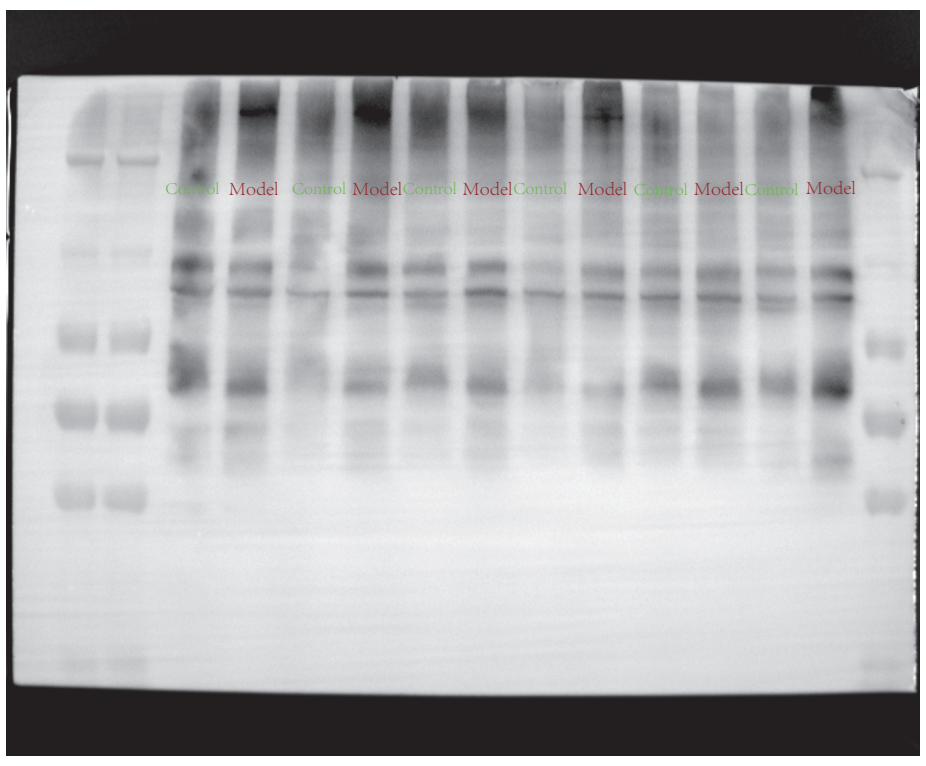

GAPDH

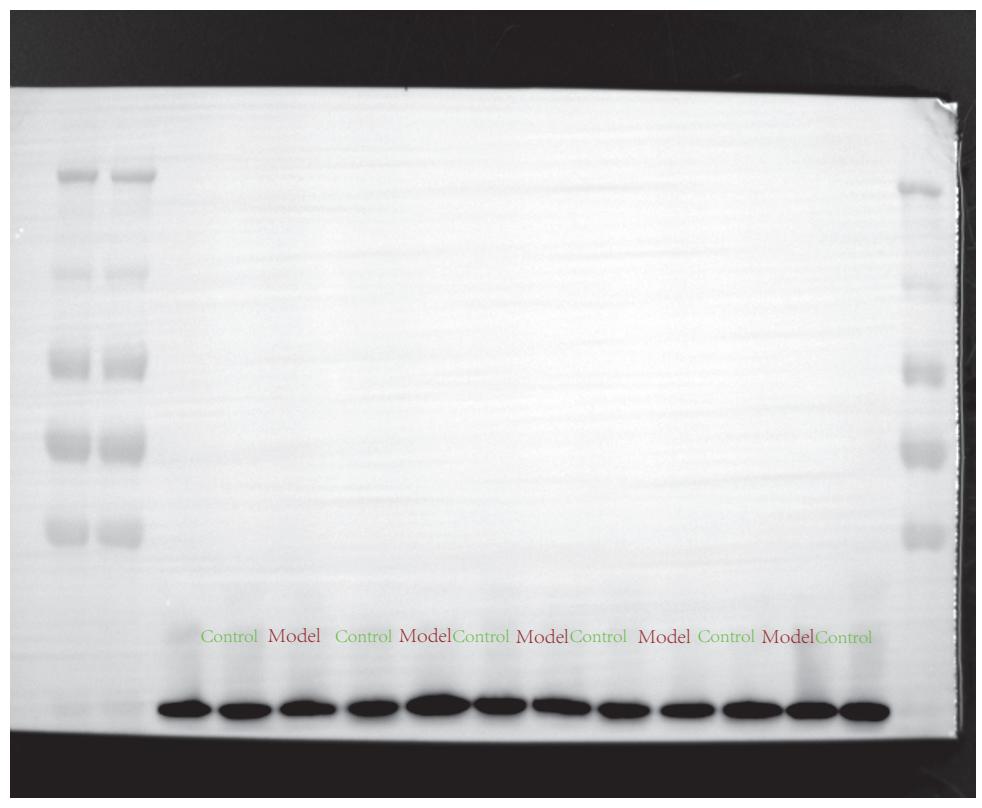

Fig 5G

PARP1

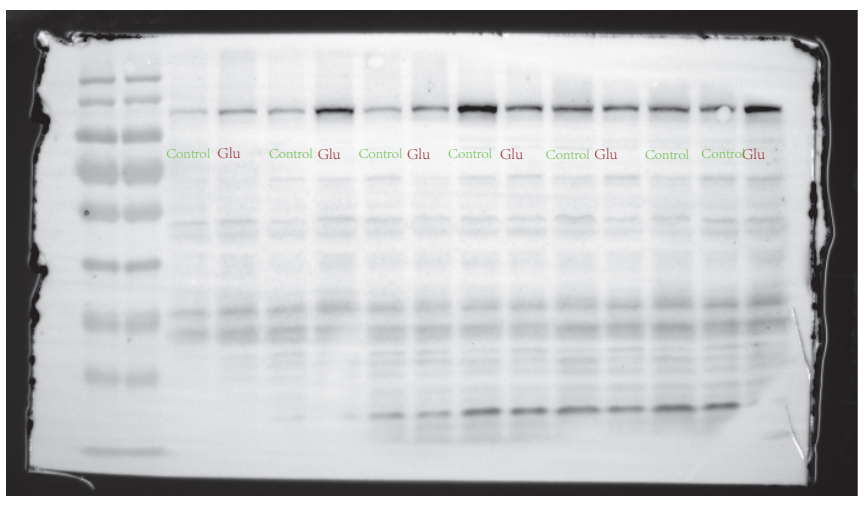

PAR

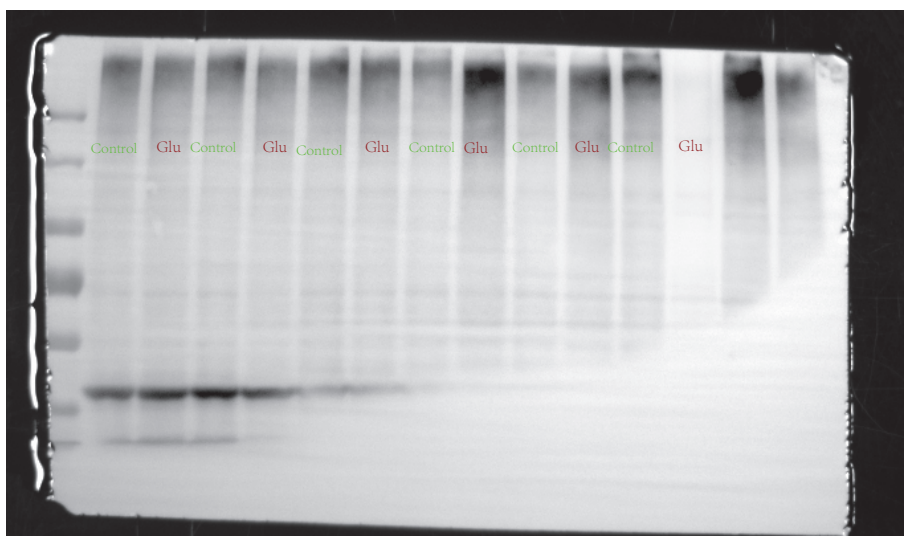

GAPDH

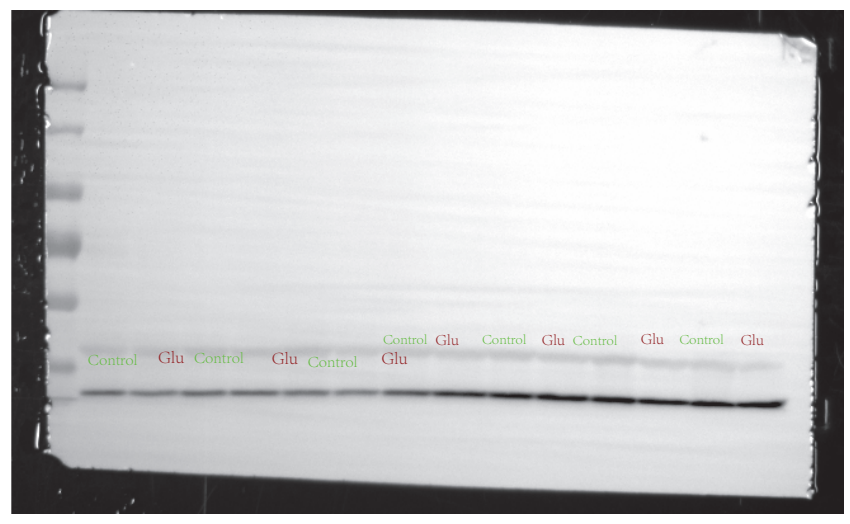

Fig5H

AIF

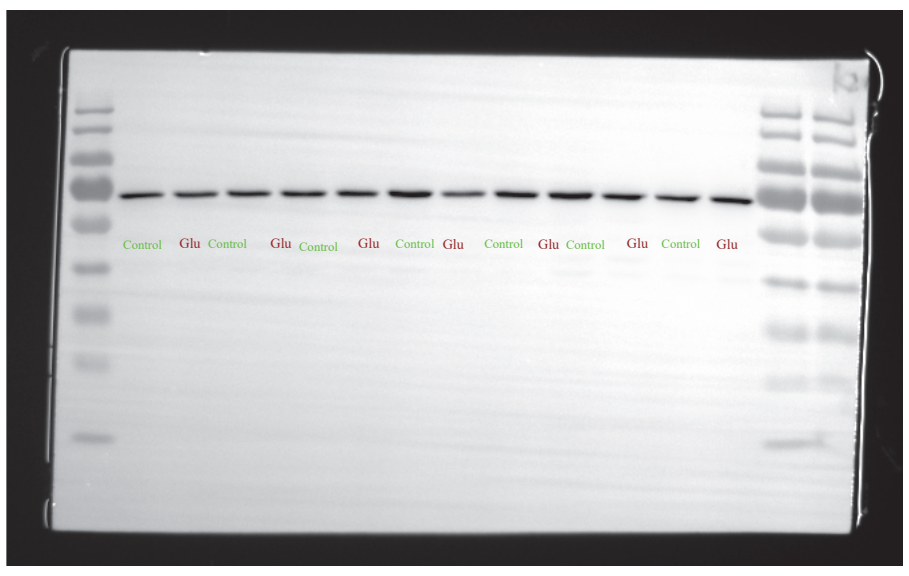

MIF

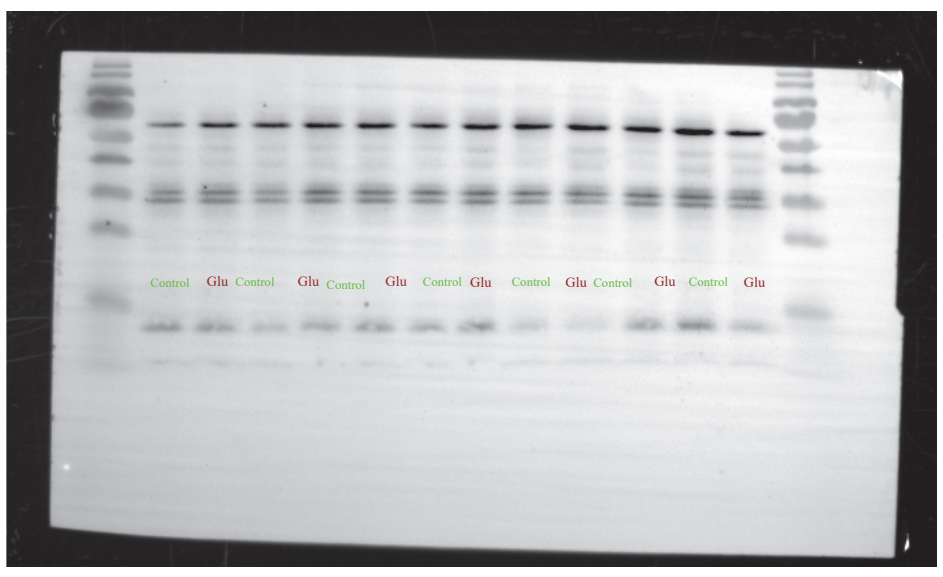

H3

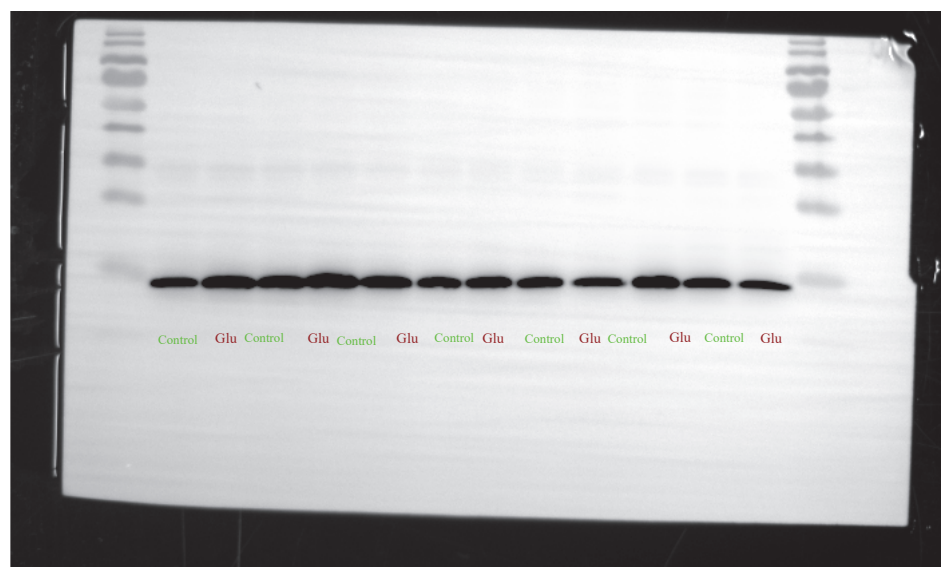

Fig 6B

PARP1

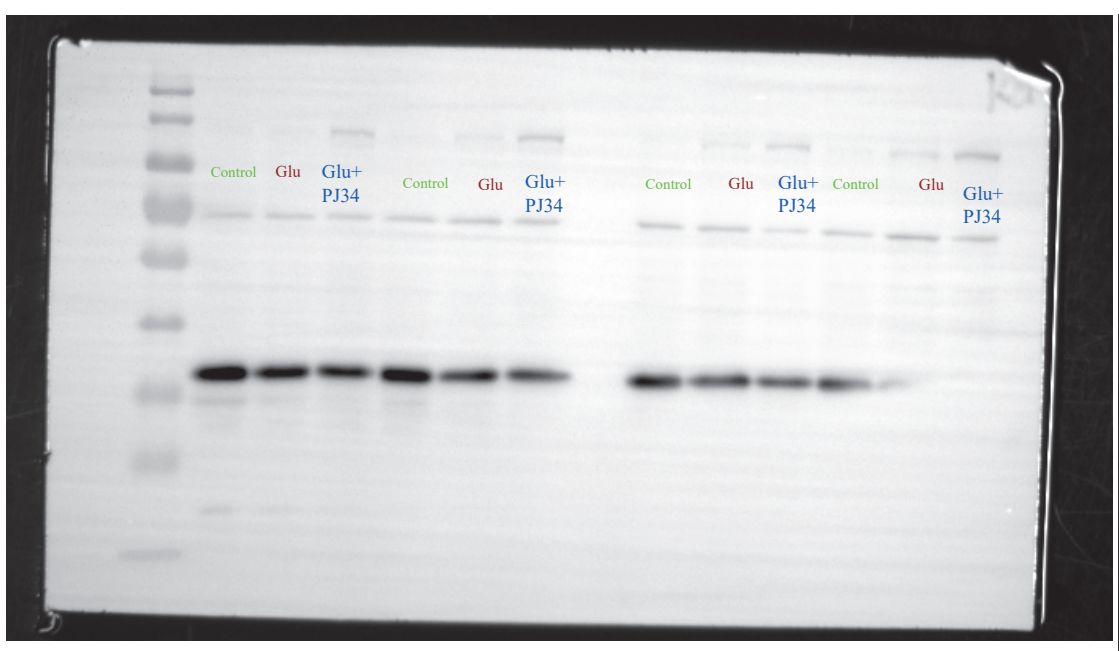

AIF

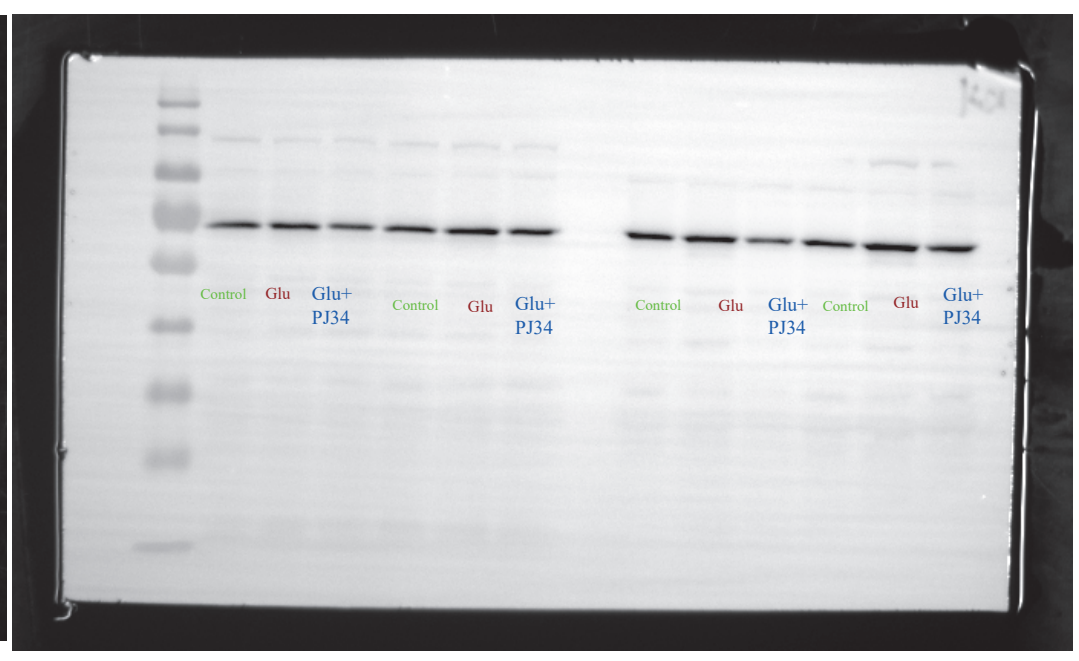

GAPDH

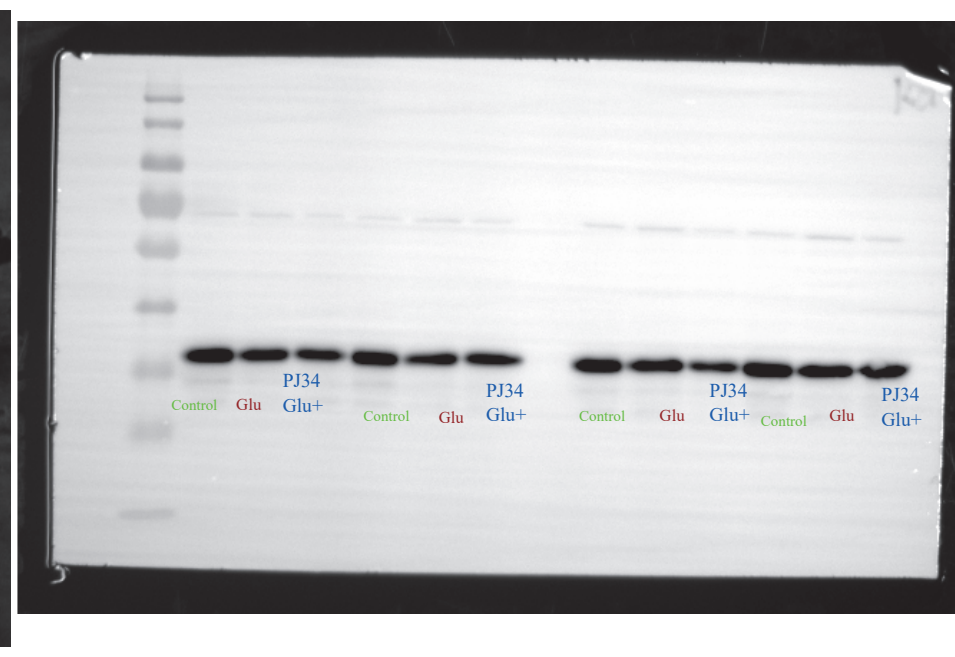

Fig 7E

PARP1

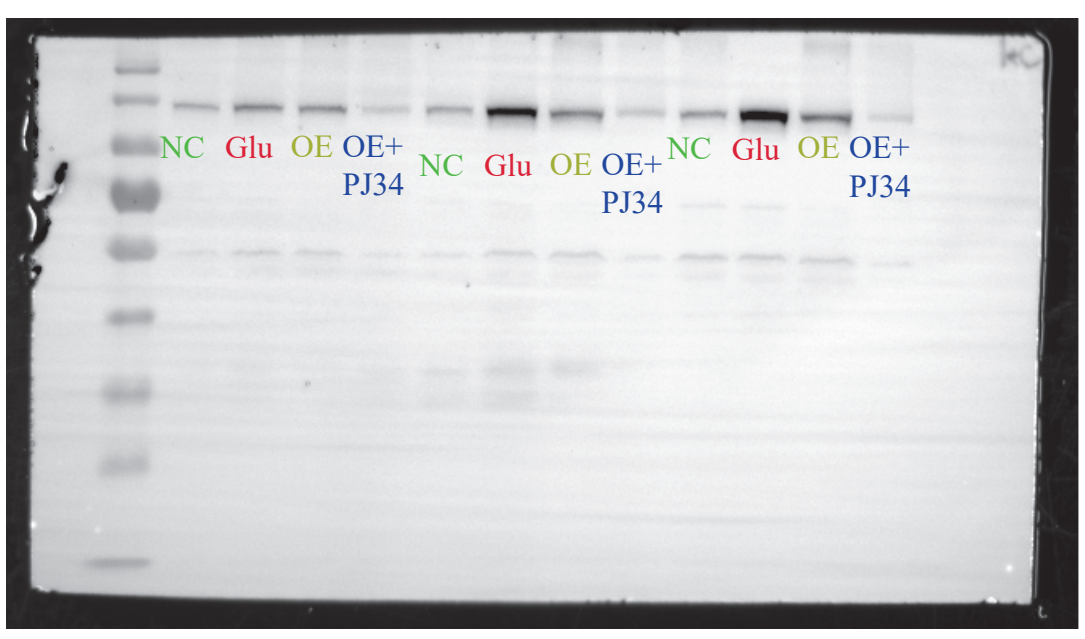

AIF

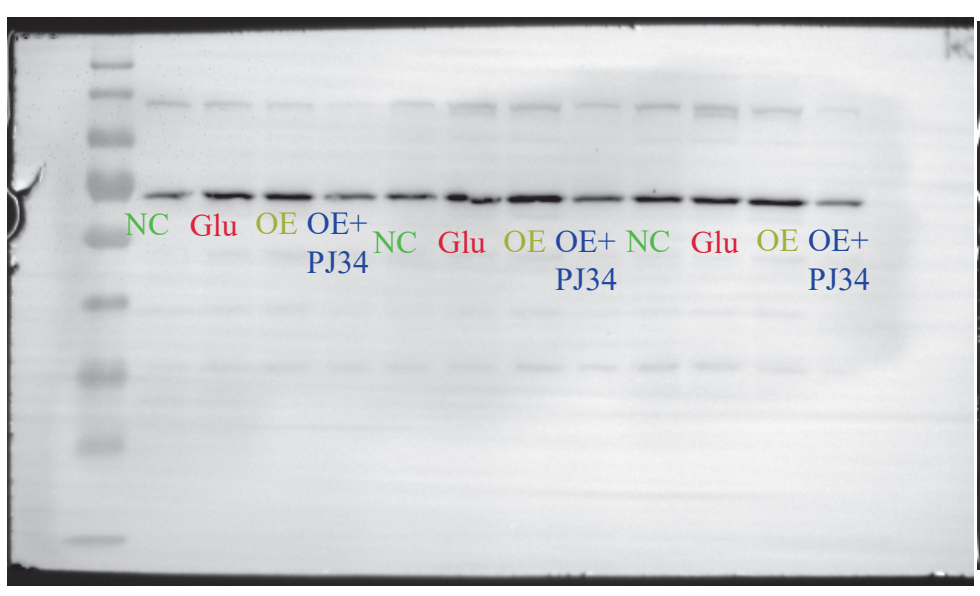

GAPDH

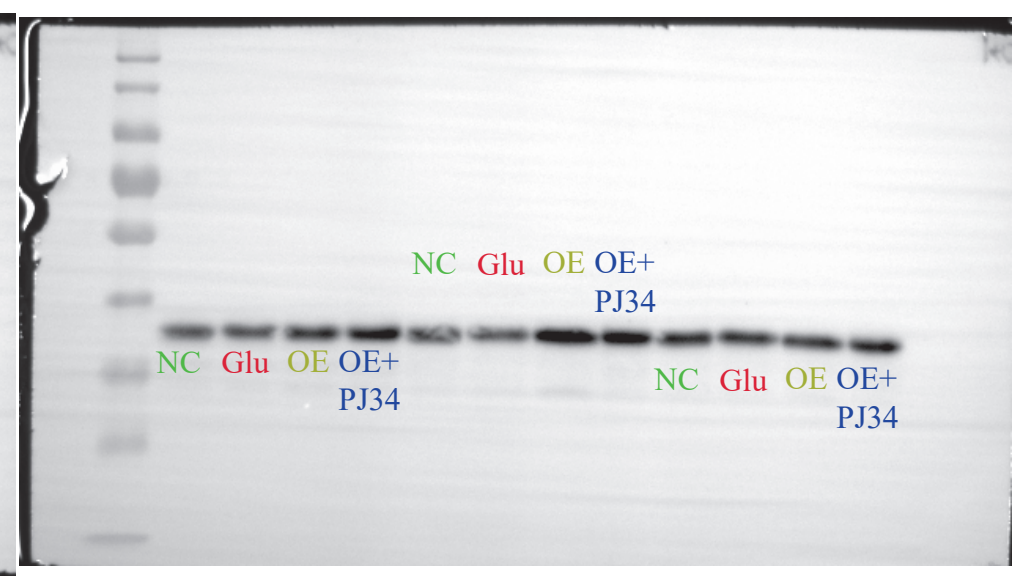

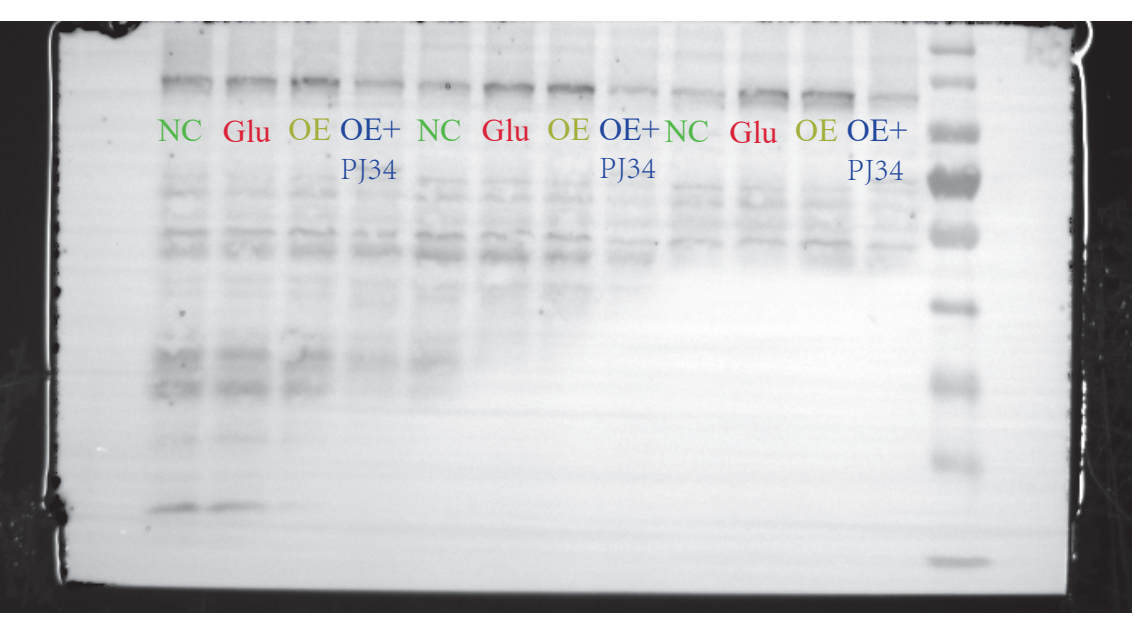

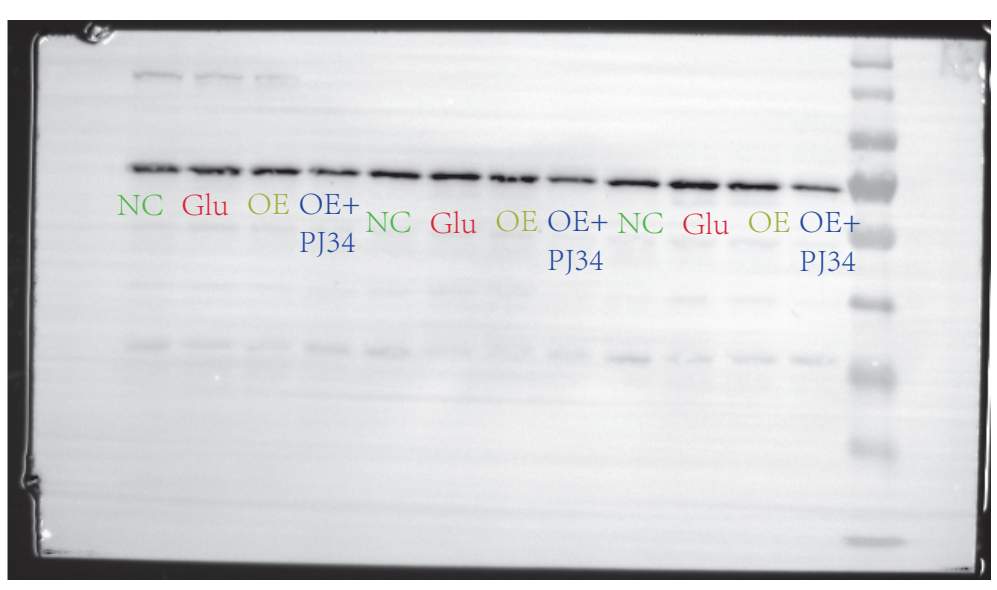

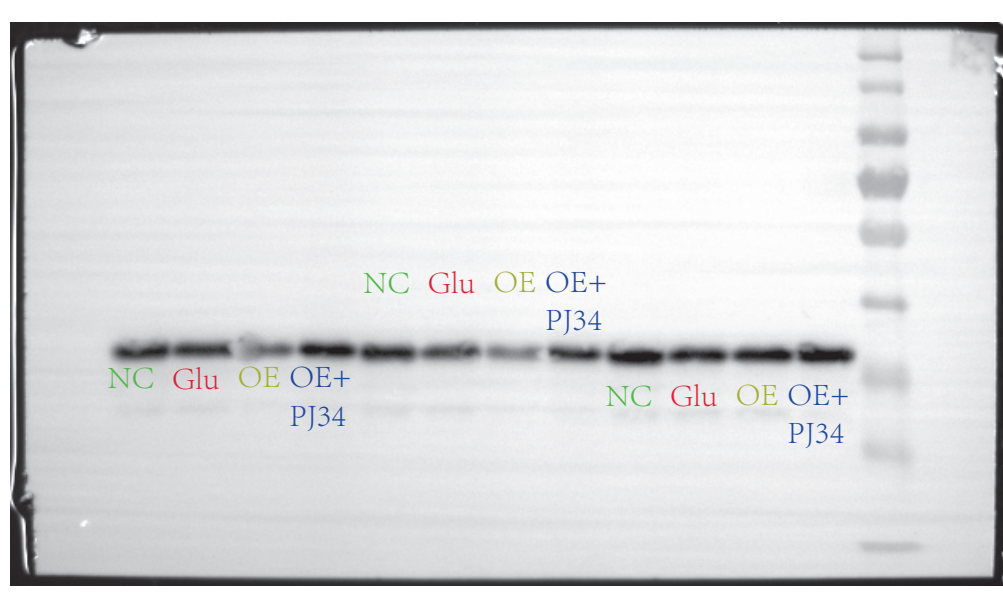

Fig S5A

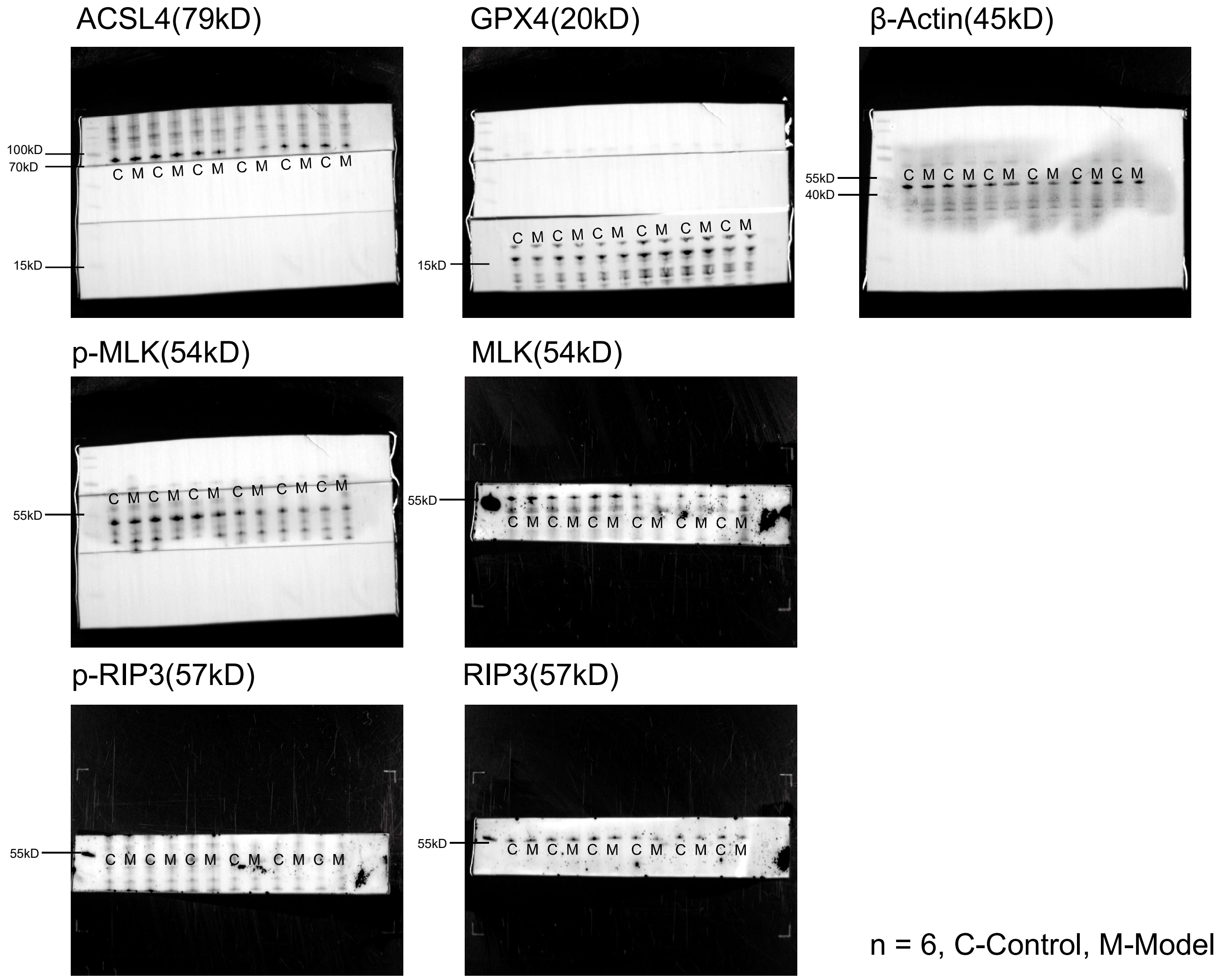

Fig S5B

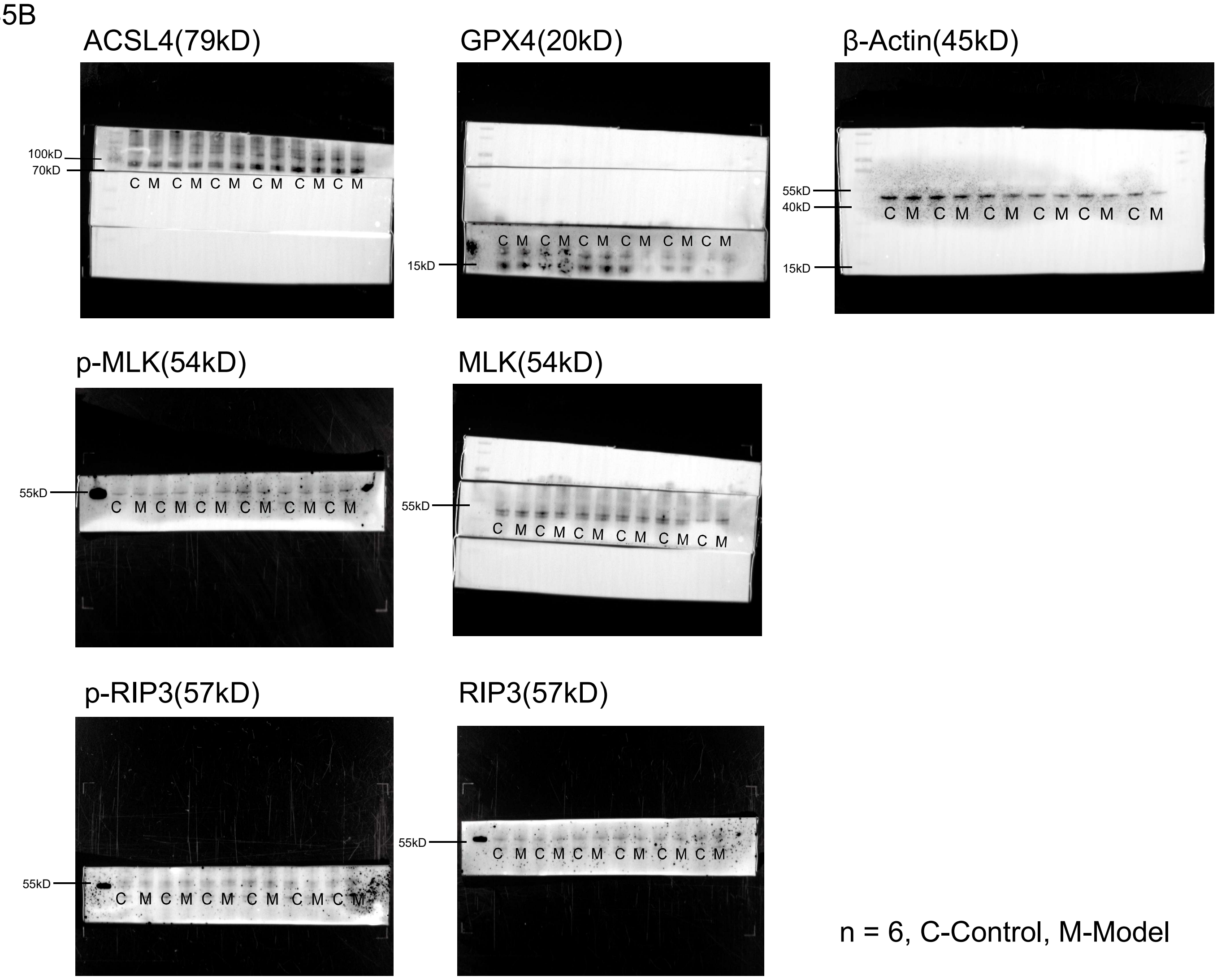

Supplement: Supplementary file 2 [file mmc2.pdf]
